# Data-driven cluster analysis and external validation identify phenotypic subgroups in renin-independent aldosteronism with differential cardiovascular risk and therapeutic implications

**DOI:** 10.3389/fendo.2025.1686480

**Published:** 2025-12-15

**Authors:** Yuqing Liu, Zhiheng Zhang, Haifeng Zhou, Yutong Yan, Mei Zhou, Cong Wang, Maoting Gao, Jun Tao, Meiling Bao, Tao Yang, Min Sun, Yuhong Yang

**Affiliations:** 1 Department of Endocrinology, The First Affiliated Hospital with Nanjing Medical University, Nanjing, China; 2 Division of Hepatobiliary and Transplantation Surgery, Department of General Surgery, Nanjing Drum Tower Hospital, Affiliated Hospital of Medical School, Nanjing University, Nanjing, China; 3 Department of Interventional Radiology, The First Affiliated Hospital with Nanjing Medical University, Nanjing, China; 4 Department of Urology, The First Affiliated Hospital with Nanjing Medical University, Nanjing, China; 5 Department of Pathology, The First Affiliated Hospital with Nanjing Medical University, Nanjing, China

**Keywords:** aldosterone, cardiovascular disease, classification, risk stratification, renin-independent aldosteronism

## Abstract

**Background:**

Renin-independent aldosterone secretion contributes to aldosteronism and heightened cardiovascular risk, but renin-independent aldosteronism is highly heterogenous. A refined classification may assist in identifying individuals with distinct cardiovascular risk profiles and guide individualized treatment strategies.

**Methods:**

Unsupervised hierarchical clustering was performed using 12 clinical parameters from patients with renin-independent aldosteronism in our registry cohort (n=404). The cluster centroids derived from the discovery cohort were fixed and applied to the Framingham Heart Study Third Generation cohort (n=417) for subject classification. The identified clusters were evaluated for their association with cardiovascular outcomes, assessed by echocardiographic parameters, serum biomarkers and cardiovascular event rates.

**Results:**

Three replicable clusters of patients with renin-independent aldosteronism were identified. Patients in cluster 2 showed the most severe metabolic abnormalities with the highest lipid and glucose levels, while patients in cluster 3 displayed the highest aldosterone levels. Both clusters 2 and 3 showed elevated baseline blood pressure and left ventricular remodeling compared with cluster 1. Cluster 2 exhibited the highest risk of cardiovascular disease, chronic heart failure and atrial fibrillation, followed by cluster 3, which showed a higher incidence of cardiovascular disease compared with cluster 1.

**Conclusions:**

We identified 3 subgroups with differing degrees of target organ damage and cardiovascular risk. Our findings establish metabolic dysfunction, rather than aldosterone excess, as a potential dominant cardiovascular risk driver in RIA patients, defining a new risk paradigm. Patients with renin-independent aldosteronism with metabolic dysfunction or high aldosterone levels may benefit from mineralocorticoid receptor antagonists with different priorities for metabolic and cardiovascular protection. This new refined classification may help tailor optimal treatment strategies for patients with heterogenous renin-independent aldosteronism.

## Introduction

Aldosterone, the predominant mineralocorticoid, is primarily regulated by renin-angiotensin II system and plays a crucial role in maintaining water and electrolyte balance by activating mineralocorticoid receptors (MRs) (1). Excessive aldosterone production that occurs independently of renin and angiotensin II, known as renin-independent aldosteronism (RIA) (2–7), leads to inappropriate activation of MRs, resulting in sodium and fluid retention, and adverse cardiorenal remodeling (8). RIA represents a pathophysiological continuum characterized by pronounced phenotypic heterogeneity. This seamless spectrum ranges from subtle autonomous aldosterone hypersecretion in normotension individuals, to mild hypertension, and ultimately to its most severe and clinically overt form with resistant hypertesion, where the majority of patients reach the diagnostic criteria for primary aldosteronism (9). Formal diagnosis of primary aldosteronism requires standardized confirmatory testing, including the commonly used saline suppression test and captopril challenge test, followed by adrenal venous sampling for subtype classification (10). In contrast, the broader RIA population encompasses all individuals with biochemical evidence of autonomous aldosterone secretion, commonly defined in previous studies (4, 5, 11) as plasma renin activity ≤ 1 µg/L/h and plasma aldosterone concentration ≥ 100 pg/mL.

Accumulating evidence have shown that compared with individuals with renin-dependent aldosteronism, those with RIA face a heightened risk of cardiovascular disease (4, 12, 13), and a higher prevalence of chronic kidney disease (5) and metabolic dysfunction-associated steatotic liver disease (6). Large-scale population studies have examined the relationship between aldosterone, renin and target organ damage, demonstrating that low renin concentrations or an elevated aldosterone-to-renin ratio are associated with incident hypertension (11, 13–16), adverse cardiac remodeling (13, 17) and increased arterial atherosclerosis (12, 13). These findings highlight the potential need for early intervention with MR antagonists in individuals with RIA to mitigate the long-term consequences of excessive aldosterone activity.

Despite these observations, the role of MR antagonists as the optimal first-line therapy for all individuals with RIA remains uncertain. A meta-analysis has demonstrated the superiority of MR antagonists over angiotensin-converting enzyme inhibitors or angiotensin receptor blockers in lowering blood pressure among hypertensive patients with low renin, who often exhibit autonomous aldosterone secretion (9, 18). Other studies have showed that diuretics and epithelial sodium channel inhibitors exhibit greater blood-pressure-lowering efficacy compared with beta-blockers and angiotensin receptor blockers (19, 20). Beyond blood pressure control, MRs activation in extra-renal tissues including the vascular and adipose tissue contributes to vascular and metabolic dysfunction, highlighting the need for therapies that address these effects (21). While angiotensin-converting enzyme inhibitors or angiotensin receptor blockers have demonstrated metabolic and cardiovascular benefits (22–26), steroidal MR antagonists have shown neutral or adverse effects on glucose and lipid metabolism (27–29). In contrast, the non-steroidal MR antagonist finerenone has been shown to reduce the hazard of diabetes in patients with heart failure (30).

The pronounced phenotypic heterogeneity of RIA poses substantial challenges for developing individualized treatment strategies. As demonstrated in other heterogeneous diseases such as type 2 diabetes and metabolic dysfunction-associated steatotic liver disease, data-driven clustering analyses can classify these patients into clinically meaningful subgroups based on multidimensional clinical features, enabling more precise risk assessment and therapeutic decision-making. In RIA, however, such approaches remain limited. Prior studies have largely relied on single biochemical thresholds (e.g., aldosterone or renin levels) or binary definitions of primary aldosteronism to differentiate cardiovascular risk, approaches that fail to capture the continuous spectrum of aldosterone excess (3, 11, 17). Moreover, no systematic effort has integrated clinical, biochemical, and metabolic variables to define distinct subgroups within the broader RIA population. Given the success of clustering-based characterizations in other heterogeneous diseases, applying an unsupervised, data-driven approach to RIA could provide a refined classification framework, identify individuals at highest cardiovascular risk, and better inform targeted use of MR antagonists. To the best of our knowledge, no prior study has applied clustering approaches to delineate clinical subphenotypes in RIA.

To fill this gap, we conducted an unsupervised, data-driven clustering analysis using 12 commonly measured variables in patients with RIA. We aimed to establish a novel RIA classification and to assess its association with specific clinical characteristics, target organ damage and long-term cardiovascular outcomes, thereby enabling the potential for early warning and precise interventions for RIA in the future.

## Materials and methods

### Study populations

**Figure 1:**
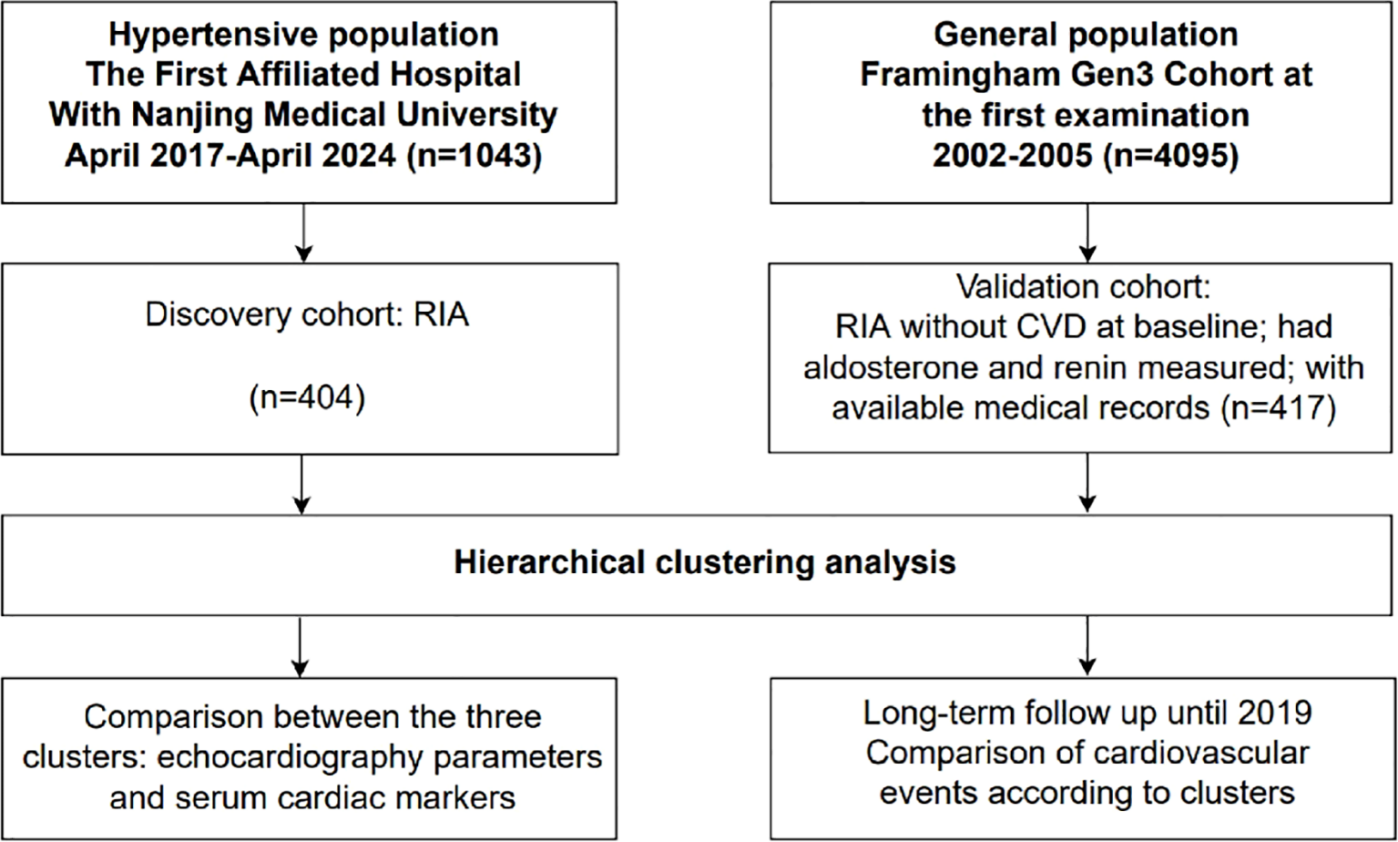
Derivation of study cohort. The study comprised 2 cohorts: the discovery cohort, from the First Affiliated Hospital with Nanjing Medical University, and the validation cohort, derived from the Framingham Heart Study Third Generation cohort. In both cohorts, renin-independent aldosteronism was defined as plasma renin activity ≤ 1 µg/L/h and plasma aldosterone concentration ≥ 100 pg/mL. CVD, cardiovascular disease; n, number.

We used data from our registry cohort and Framingham Heart Study Third Generation (Gen III) cohort. The study flow chart of participant selection was shown in **Figure 1**.

In our registry cohort, all patients were screened for endocrine hypertension at the First Affiliated Hospital With Nanjing Medical University, Nanjing, China (31). We included patients who met all of the following criteria: plasma renin activity ≤ 1 µg/L/h, plasma aldosterone concentrations ≥ 100 pg/mL, and available medical records including parameters used for clustering, echocardiographic data and serum cardiac parameters at study entry.

The Framingham Heart Study Gen III cohort is a longitudinal cardiovascular epidemiological study with a previously published study design (32–34). It began in 2002, and was funded by the National Institutes of Health and administered by Boston University. We included patients who attended the Gen III cohort examination 1 (2002-2005) and were followed up until December 31, 2019, and met all of the following criteria: no history of cardiovascular disease, plasma renin activity ≤ 1 µg/L/h, plasma aldosterone concentrations ≥ 100 pg/mL, and available medical records including parameters used for clustering at study entry.

### Physical examination and laboratory measurement

In our registry cohort, all antihypertensive medications known to interfere with renin-angiotensin-aldosterone system activity (including diuretics, beta-blockers, angiotensin converting enzyme inhibitors, and angiotensin-1 receptor blockers) were withdrawn, or replaced with calcium channel blockers or alpha-adrenergic blockers for 2–4 weeks before blood samples collection. Hypokalemia was corrected to minimize its impact on aldosterone and renin measurements. Blood samples were drawn following an overnight fasting period (≥ 8 hours) with patients in the upright position. Routine biochemical measurements were conducted using enzymatic methods on a chemistry analyzer (Au5800, Olympus Medical Engineering Company). Plasma aldosterone concentrations were measured by a chemiluminescent assay (Mindray), with intra- and inter-batch coefficients of variation ranging from 3.26% to 5.69% and 5.61% to 7.30%, respectively. Plasma renin activity was quantified via radioimmunoassay (Beijing North Institute of Biotechnology), with intra-batch coefficients of variation < 10% and inter-batch coefficients of variation < 15%. Obesity was defined according to the Chinese guidelines for management of obesity as a body mass index (BMI) ≥ 28 kg/m^2^.

In Gen III cohort, physical examination procedures and laboratory measurements have been described previously (35, 36). Venipuncture was performed on study participants in a supine position after an overnight fast using standard techniques. We reviewed medical history and collected anthropometric data including BMI, systolic and diastolic blood pressure. Plasma aldosterone concentrations were measured using an enzyme-linked immunosorbent assay (ALPCO Diagnostics), and plasma renin activity was measured with a radioimmunoassay (DiaSorin). In both cohorts, the estimated glomerular filtration rate was calculated using Chronic Kidney Disease Epidemiology Collaboration (37). Obesity was defined as a BMI ≥ 30 kg/m^2^ according to the European Society of Endocrinology Clinical Practice Guideline (38).

### Definitions of RIA and primary aldosteronism subtyping

In our registry and Gen III cohorts, RIA was defined in accordance with previous studies (4, 5, 11) as plasma renin activity ≤ 1 µg/L/h and plasma aldosterone concentration ≥ 100 pg/mL. Within the discovery cohort, patients who met the RIA criteria underwent further evaluation for primary aldosteronism. The diagnosis of primary aldosteronism required a positive screening test (aldosterone-to-renin ratio ≥ 20 pg/dL per µg/L/h) together with at least one positive confirmatory test (saline infusion test or captopril challenge test) (10). Patients with confirmed primary aldosteronism were further subtyped as follows: unilateral primary aldosteronism was diagnosed when patients met any of the following criteria: (1) a lateralization ratio ≥ 2.0 following successful catheterization (selectivity index ≥ 2.0) during adrenal venous sampling; (2) complete biochemical success at ≥ 6 months after adrenalectomy; or (3) decision to bypass adrenal venous sampling based on strong clinical and radiological evidence; bilateral primary aldosteronism was diagnosed when patients met either of the following criteria: (1) a lateralization ratio < 2.0 following successful adrenal venous sampling; or (2) a Japan Primary Aldosteronism Study score ≥ 8 (39), predicting a low likelihood of unilateral disease.

### Assessment of outcomes

In our registry cohort, we assessed cardiovascular health across RIA clusters using echocardiographic parameters, serum cardiac markers and arterial vascular parameters. The existence of cardiovascular disease was confirmed after rigorous review of hospitalization records, outpatient clinic visits, and diagnostic test results. It was confirmed when meeting the following conditions: (1) coronary heart disease: documented myocardial infarction (confirmed by characteristic cardiac enzyme elevation and electrocardiographic changes) or coronary revascularization procedures (percutaneous coronary intervention or coronary artery bypass grafting); (2) heart failure: clinical diagnosis of congestive heart failure requiring hospitalization or consistent outpatient management, supported by echocardiographic evidence of systolic or diastolic dysfunction; (3) stroke: acute cerebrovascular event confirmed by neuroimaging (computed tomography or magnetic resonance imaging), including both ischemic and hemorrhagic strokes; (4) transient ischemic attack: documented neurological deficits resolving within 24 hours with corresponding neuroimaging findings.

In the Gen III cohort, cardiovascular disease event was defined by the occurrence of a composite endpoint according to validated Framingham Heart Study criteria (34), comprising coronary heart disease, congestive heart failure, cerebrovascular events (including ischemic stroke, hemorrhagic stroke, and transient ischemic attack) and peripheral artery disease (intermittent claudication) (40, 41). Outcomes were ascertained through medical histories, physical examinations, hospitalization records, and communication with participants’ physicians (42). Specific component definitions were as follows: (1) coronary heart disease: coronary death, myocardial infarction, or hospitalized coronary insufficiency (42); (2) stroke: events confirmed by at least two neurologists via medical record review, including definite cerebrovascular accident, atherothrombotic brain infarction, cerebral embolism, intracerebral hemorrhage, or subarachnoid hemorrhage (43); (3) congestive heart failure events were adjudicated by an independent endpoint committee according to the Framingham criteria (44), requiring either: a. ≥ 2 major clinical criteria (e.g., paroxysmal nocturnal dyspnea, jugular venous distension), or b. 1 major criterion + ≥ 2 minor criteria (e.g., ankle edema, dyspnea on exertion); (4) atrial fibrillation was diagnosed if at least 2 cardiologists verified the rhythm abnormality on an electrocardiogram, including Holter electrocardiograms, telemetry or other monitoring data (45, 46).

In both cohorts, left ventricular hypertrophy was defined by a left ventricular mass index ≥115 g/m^2^ in men and ≥95 g/m^2^ in women, following the recommendation of the American Society of Echocardiography (47).

### Follow-up duration and completeness

Our analysis included Gen III cohort participants from the baseline examination (2002-2005) who had complete follow-up through the study end date. The Framingham Heart Study is characterized by its excellent participant retention, historically achieving a 99% retention rate (40). Participants in this study were followed for a median of 5543 days [4899–6033 days] until December 31, 2019.

### Echocardiographic examination

In our registry cohort, all participants underwent 2-dimensional transthoracic echocardiography performed by experienced sonographers using a Phillips EPIQ CVx ultrasound system. Left ventricular mass and left ventricular mass index were calculated using the Devereux and Reichek formula (48). In the Gen III cohort, standardized 2-dimensional echocardiography with color flow and tissue Doppler imaging was performed by experienced sonographers using a Hewlett-Packard Sonos 5500 Ultrasound machine (Phillips Healthcare, Andover, MA) (49). Experienced readers evaluated the digitized echocardiographic images (DICOM format) utilizing Digiview System Software (version 3.7.9.3, Digisonic Inc., Houston, TX, USA).

### Cluster analysis and validation

Our registry cohort (hereafter named as discovery cohort) served as the discovery cohort for establishing unsupervised clustering, and the Gen III cohort (hereafter named as validation cohort) was used as the validation cohort to assess the reproducibility of these clusters and their association with cardiovascular risk. Missing data in the discovery cohort were imputed using multiple imputation by the R package “mice” before clustering analysis, while the validation cohort had complete data and did not require imputation. In the discovery cohort, the proportion of missing data for the 12 clustering variables was generally low, with fasting insulin having the highest proportion at 2.48% (10 missing values), followed by cortisol at 0.50% (2 missing values), while the remaining 10 variables had complete data (0% missing) (**Supplementary Figure S1**). The overall missing rate across all clustering variables in the discovery cohort was 0.25%. We employed the following multiple imputation parameters: 5 imputed datasets (m=5), maximum of 10 iterations, predictive mean matching method, and random seed 12345 to ensure reproducibility. The selection of the 12 clustering parameters was based on their established pathophysiological relevance to RIA. Age at diagnosis, sex, and BMI were included as core demographic and adiposity modifiers. Systolic and diastolic blood pressure were incorporated as principal hemodynamic consequences of aldosterone excess. Plasma renin activity and plasma aldosterone concentrations represented the defining biochemical axis of RIA. Metabolic markers (fasting blood glucose, triglycerides, total cholesterol and fasting insulin) were selected due to the established comorbidity between metabolic dysfunction and aldosterone excess. Cortisol was included to account for its co-secretion and influence on associated comorbidities. Overall, correlations between the selected parameters were weak, as visualized in the correlation matrix (**Supplementary Figure S2**). The strongest correlation identified was a moderate positive correlation between systolic blood pressure and diastolic blood pressure (r = 0.56). Hierarchical clustering using Ward’s minimum variance method was applied to the discovery cohort based on the distance matrix of 12 clinical parameters. The optimal number of clusters was determined using the cubic clustering criterion, yielding a 3-cluster solution. For external validation, representative subjects were selected from each cluster in the discovery cohort based on proximity to the cluster centroid in the multidimensional feature space. Each subject in the validation cohort was then assigned to the cluster of its nearest representative subject from the discovery cohort, using Euclidean distance, without performing *de novo* clustering in the validation cohort.

### Statistical analysis

Data were analyzed with the use of SPSS version 27.0, GraphPad Prism version 10.1 and R software version 4.0.3. Continuous variables were expressed as mean (standard deviation) for normally distributed data, median (interquartile range) for non-normally distributed data, while categorical variables were presented as numbers and proportions. Normality was assessed using the Kolmogorov-Smirnov test. Differences in continuous variables were evaluated using ANOVA followed by Bonferroni tests or Kruskal-Wallis tests followed by pairwise comparisons. Chi-square and Fisher’s exact tests were used to compare categorical data. Distinct regression models were employed for each cohort based on their study designs to assess the cluster-cardiovascular outcome association. In the discovery cohort (cross-sectional analysis), multivariable logistic regression was used to assess the odds of prevalent cardiovascular disease across clusters (reference: cluster 1), adjusted for smoking, alcohol use, statin use, and diabetes treatment. In the validation cohort (longitudinal analysis), Cox proportional hazards models were used to estimate risks of incident cardiovascular events (reference: cluster 1), with adjustment for the same covariates. Kaplan-Meier curves and log-rank tests were used for visualizing and comparing cumulative event rates. Results are reported as hazard ratios with 95% confidence intervals. A *P* value < 0.05 was considered as statistically significant.

## Results

### Clinical characteristics of study populations

A total of 404 patients and 417 patients with RIA, derived from the discovery and validation cohorts, respectively, were included in **Figure 1**. Patient characteristics were shown in **Supplementary Table S1**. RIA patients in the validation cohort were younger, and had higher BMI, fasting blood glucose and total cholesterol, and lower systolic blood pressure, diastolic blood pressure, plasma aldosterone concentrations and aldosterone-to-renin ratio, and proportion of diabetes compared with those in the discovery cohort (all, *P* < 0.05) (**Supplementary Table S1**).

### Clinical characteristics of distinct clusters

**Figure 2:**
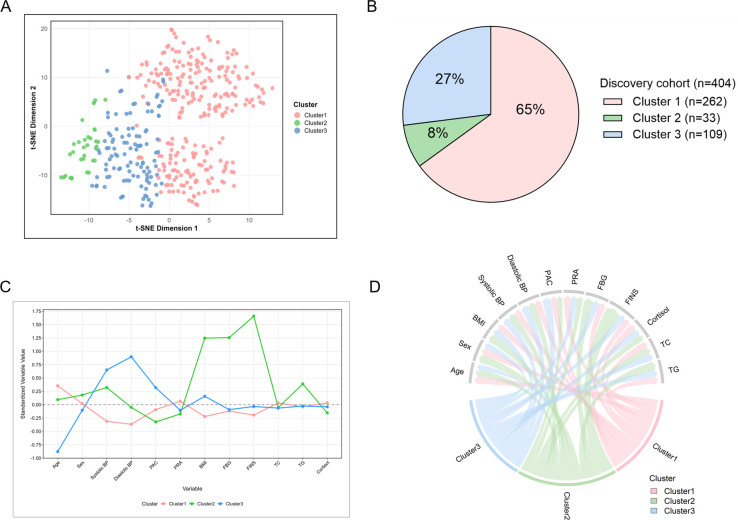
Visualization of clustering results and patient distribution in the discovery cohort. **(A)** t-Distributed Stochastic Neighbor Embedding (t-SNE) plot visualizing high-dimensional data. **(B)** Patient distribution across clusters. **(C)** Standardized values (z-scores) of selected variables by cluster. All continuous variables were scaled (mean = 0, SD = 1). **(D)** The ribbons connect an individual cluster to a variable if the group mean is greater or less than the overall mean for the entire cohort. BMI, body mass index; BP, blood pressure; FBG, fasting blood glucose; FINS, fasting insulin; PAC, plasma aldosterone concentration; PRA, plasma renin activity; RIA, renin-independent aldosteronism; TC, total cholesterol; TG, triglycerides.

**Figure 3:**
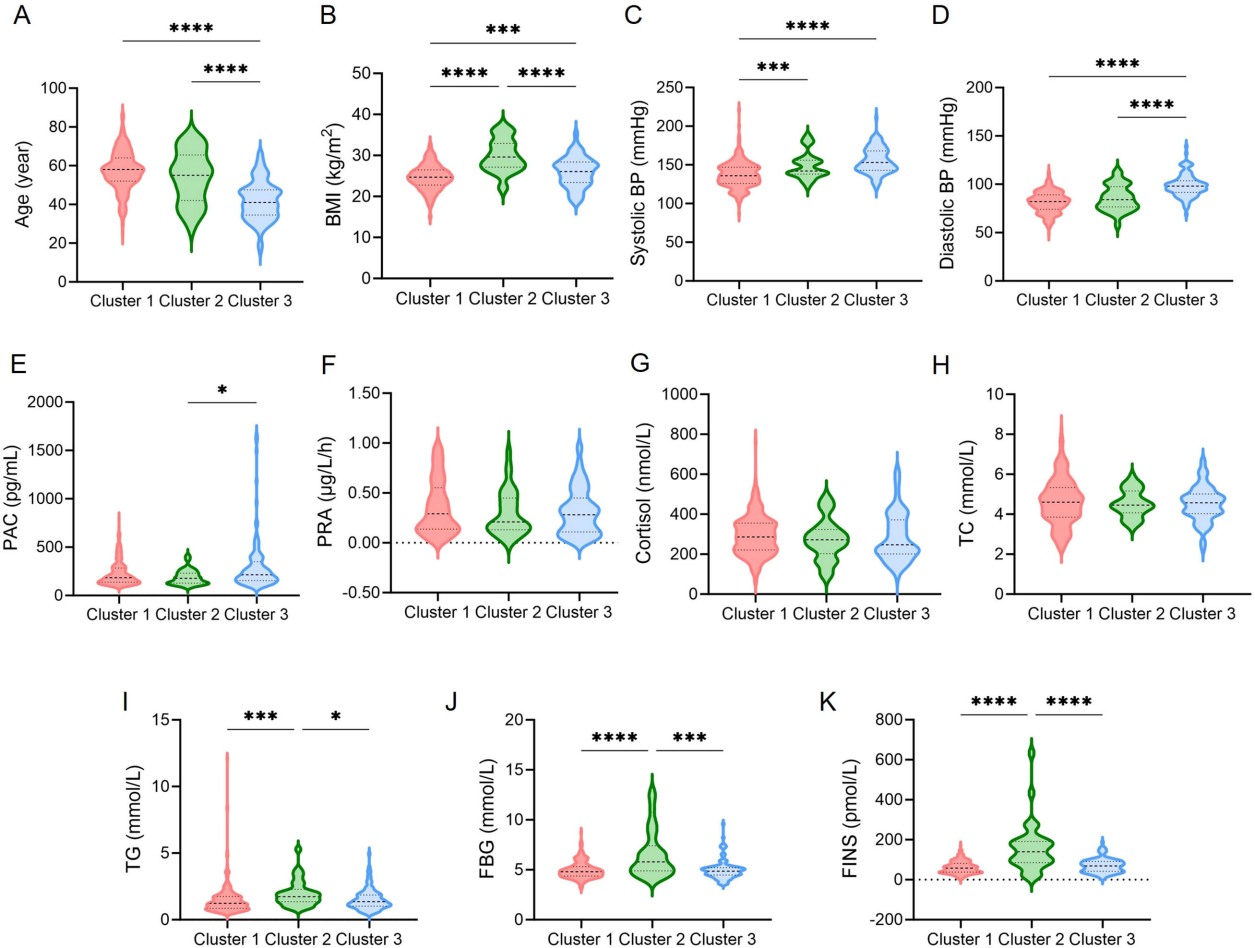
Clinical characteristics of patients with RIA stratified by different clusters in the discovery cohort. The distribution of **(A)** age, **(B)** BMI, **(C)** systolic blood pressure, **(D)** diastolic blood pressure, **(E)** plasma aldosterone concentration, **(F)** plasma renin activity, **(G)** cortisol, **(H)** total cholesterol, **(I)** triglycerides, **(J)** fasting blood glucose, and **(K)** fasting insulin in different clusters in the discovery cohort. BMI, body mass index; BP, blood pressure; FBG, fasting blood glucose; FINS, fasting insulin; h, hour; PAC, plasma aldosterone concentration; PRA, plasma renin activity; RIA, renin-independent aldosteronism; TC, total cholesterol; TG, triglycerides. ^*^P < 0.05; ^***^P < 0.001; ^****^P < 0.0001.

A dendrogram showing the patient clustering process is presented in **Supplementary Figure S3**. We identified 3 major clusters in the discovery cohort data. To visualize these clusters and explore their distinctions, we generated 2D representations using t-distributed stochastic neighbor embedding (t-SNE) (**Figure 2**). The proportions of the 3 clusters identified in the discovery and validation cohorts are shown in **Figure 2** and **Supplementary Figure S4B**. The distinct variables of 3 clusters are depicted in **Figure 2** and 2D. The baseline clinical characteristics of patients stratified by these clusters are shown in **Figure 3** and **Supplementary Table S2**. Patients in cluster 1 (n=262, 64.9%) were the oldest (*P*<0.001), and displayed the lowest level of systolic (*P*<0.001) and diastolic blood pressure (*P*<0.001), and the least severe metabolic dysfunction compared with the other 2 clusters. Cluster 2 (n=33, 8.2%) comprised patients with the most pronounced metabolic abnormalities, characterized by the highest BMI (*P*<0.001), triglycerides (*P* = 0.001) and fasting blood glucose (*P*<0.001), and the highest proportion of obesity (*P*<0.001) and diabetes (*P* = 0.002), leading to the greatest use of statins (*P* = 0.001) and antidiabetic therapy (*P* = 0.002). Patients in cluster 3 (n=109, 27.0%) showed the highest plasma aldosterone concentrations (*P* = 0.009) and aldosterone-to-renin ratio (*P* = 0.042). The proportion of confirmed primary aldosteronism (*P* = 0.047) was highest in cluster 3, whose patients also showed a high prevalence of unilateral primary aldosteronism (87% of 79, *P* = 0.173) (**Supplementary Figures S5, S6**).

### Cardiovascular disease incidences and cardiac parameters of distinct clusters

Cluster 1 was associated with the least severe abnormalities of cardiac structure, while patients in cluster 2 and cluster 3 displayed a comparably elevated echocardiographic parameters indicative of increased left ventricular size and dysfunction, including left ventricular mass (*P* = 0.001), left ventricular end-systolic (*P* = 0.032) and end-diastolic diameter (*P* = 0.012), and interventricular septal thickness (*P* = 0.017). Patients in cluster 2 exhibited the highest levels of arterial stiffness markers, including lipoprotein-associated phospholipase 2 (*P* = 0.034) and mean brachial-ankle pulse wave velocity (*P* = 0.027). The lowest level of estimated glomerular filtration rate was identified in the cluster 1 (*P*<0.001) (**Table 1**).

**Table 1:** Parameters related to target organ damage in different clusters of patients with RIA in the discovery cohort.

Variables	Total (n=404)	Cluster 1 (n=262)	Cluster 2 (n=33)	Cluster 3 (n=109)	Overall *P* value	Pairwise Comparison (*P* value)		
						Cluster 1 vs Cluster 2	Cluster 1 vs Cluster 3	Cluster 2 vs Cluster 3
Echocardiography parameters (n=286)								
Aod, mm	30.00 (28.00-32.00)	30.50 (29.00-32.00)	31.00 (27.75-33.25)	30.00 (27.00-32.00)	0.593	NA	NA	NA
LAD, mm	37.14 ± 4.31	37.00 ± 4.35	39.37 ± 3.50	36.63 ± 4.28	0.015	0.022	1.000	0.015
LVDd, mm	49.00 (46.00-50.00)	47.00 (45.00-50.00)	49.00 (47.75-54.00)	50.00 (49.00-52.00)	0.012	0.095	0.046	1.000
LVDs, mm	31.00 (30.00-33.00)	31.00 (29.75-32.00)	32.00 (30.00-35.25)	32.00 (31.00-33.00)	0.032	0.101	0.171	1.000
IVS, mm	11.00 (10.00-12.00)	10.00 (10.00-11.00)	11.00 (10.00-12.25)	11.00 (10.00-11.00)	0.017	0.123	0.058	1.000
LVPW, mm	10.00 (10.00-11.00)	10.00 (9.75-10.00)	10.00 (10.00-12.00)	10.00 (10.00-11.00)	0.023	0.086	0.133	1.000
LVM, g	181.94 (158.21-213.88)	164.45 (151.91-198.70)	194.02 (168.73-240.29)	194.38 (169.85-220.76)	0.001	0.016	0.018	1.000
LVMI, g/m^2^	103.32 (92.19-118.19)	98.99 (89.59-112.36)	104.49 (88.52-126.06)	104.30 (95.84-126.97)	0.236	NA	NA	NA
LVH, %	143 (50.0%)	93 (48.4%)	15 (55.6%)	35 (52.2%)	0.721	NA	NA	NA
RWT	0.42 (0.39-0.45)	0.43 (0.39-0.45)	0.42 (0.39-0.46)	0.41 (0.38-0.44)	0.575	NA	NA	NA
RAD, mm	34.00 (31.00-36.00)	33.00 (30.00-35.25)	34.50 (30.75-37.00)	34.00 (32.00-36.00)	0.618	NA	NA	NA
RVDd, mm	33.51 ± 3.07	33.33 ± 3.05	33.57 ± 2.58	34.02 ± 3.26	0.328	NA	NA	NA
FS, %	34.83 ± 2.10	34.82 ± 2.17	34.64 ± 1.80	34.95 ± 2.02	0.798	NA	NA	NA
LVEF, %	63.89 ± 2.93	63.90 ± 3.05	63.54 ± 2.43	64.00 ± 2.78	0.788	NA	NA	NA
E1, cm/s	74.28 ± 19.42	74.23 ± 19.38	78.94 ± 17.99	72.67 ± 20.04	0.403	NA	NA	NA
A1, cm/s	81.82 ± 17.64	83.07 ± 18.70	89.08 ± 17.19	75.57 ± 12.35	0.001	0.330	0.009	0.004
Spetal e’, cm/s	7.03 ± 2.04	7.08 ± 1.96	6.81 ± 2.12	6.98 ± 2.23	0.813	NA	NA	NA
Lateral e’, cm/s	9.52 ± 2.70	9.36 ± 2.44	10.00 ± 3.06	9.80 ± 3.22	0.357	NA	NA	NA
E/A ratio	0.90 (0.70-1.20)	0.90 (0.70-1.20)	0.90 (0.78-1.13)	0.90 (0.80-1.20)	0.585	NA	NA	NA
E/e’ ratio	8.90 (7.80-10.70)	8.80 (7.78-10.80)	8.85 (7.90-12.20)	8.30 (6.30-12.10)	0.568	NA	NA	NA
Serum cardiac markers								
NT-proBNP, pg/mL (n=313)	69.78 (36.13-124.50)	62.70 (35.11-111.03)	55.38 (28.17-179.68)	88.81 (19.93-137.60)	0.209	NA	NA	NA
cTnT, ng/L (n=233)	7.54 (5.74-11.61)	7.10 (5.29-9.50)	7.19 (5.52-10.08)	9.02 (5.00-12.70)	0.763	NA	NA	NA
Lp-PLA_2_, ng/mL (n=289)	112.00 (100.00-174.00)	101.00 (100.00-131.00)	164.50 (118.75-232.50)	114.00 (100.00-175.00)	0.034	0.036	0.713	0.308
Cardiovascular outcome (n=404)								
CVD, %	43 (10.6%)	22 (8.4%)	8 (24.2%)	13 (11.9%)	0.025	0.010	0.289	0.096
Arterial vascular parameters (n=258)								
Right ABI	1.18 (1.12-1.23)	1.19 (1.13-1.23)	1.19 (1.11-1.28)	1.21 (1.09-1.28)	0.731	NA	NA	NA
Left ABI	1.17 (1.11-1.22)	1.17 (1.13-1.20)	1.18 (1.13-1.26)	1.21 (1.12-1.26)	0.216	NA	NA	NA
Mean ABI	1.18 (1.12-1.22)	1.19 (1.13-1.22)	1.18 (1.13-1.27)	1.21 (1.11-1.27)	0.493	NA	NA	NA
Maximum ABI	1.20 ± 0.09	1.20 ± 0.09	1.19 ± 0.14	1.21 ± 0.08	0.601	NA	NA	NA
Right baPWV, cm/s	1572.50 (1417.00-1779.00)	1495.50 (1409.25-1727.25)	1692.00 (1467.75-2135.50)	1483.00 (1374.00-1713.00)	0.114	NA	NA	NA
Left baPWV, cm/s	1645.63 ± 294.38	1644.01 ± 279.57	1766.56 ± 411.13	1605.67 ± 271.11	0.064	NA	NA	NA
Mean baPWV, cm/s	1629.79 ± 304.40	1630.09 ± 272.30	1769.28 ± 385.52	1579.25 ± 331.63	0.027	0.097	0.715	0.022
Maximum baPWV, cm/s	1667.07 ± 296.61	1662.85 ± 280.68	1808.48 ± 403.77	1625.84 ± 277.52	0.029	0.065	1.000	0.025
Renal function (n=404)								
Cr, µmol/L	73.52 ± 23.10	73.97 ± 24.83	69.12 ± 18.62	73.77 ± 19.79	0.520	NA	NA	NA
eGFR, mL/min/1.73m^2^	93.85 ± 18.64	90.81 ± 17.24	95.43 ± 18.53	100.66 ± 20.15	<0.001	0.508	<0.001	0.446
CKD, %	25 (6.2%)	19 (7.3%)	0 (0%)	6 (5.5%)	0.541	NA	NA	NA

Quantitative normally distributed variables are expressed as means with standard deviations and non-normally distributed variables are shown as medians and interquartiles. Categorical variables are presented as absolute numbers and percentages. P values are calculated using Chi-square and Fisher’s exact tests or ANOVA followed by Bonferroni tests or Kruskal-Wallis tests followed by pairwise comparisons as appropriate. P<0.05 was considered significant. A, late mitral diastolic flow velocity; ABI, ankle brachial index; Aod, aortic root diameter; baPWV, brachial-ankle pulse wave velocity; CKD, chronic kidney disease; Cr, creatinine; cTnT, cardiac troponin T; CVD, cardiovascular disease; E, early mitral diastolic flow velocity; e’, velocity of mitral annular lateral and interwall motion; eGFR, estimated glomerular filtration rate (Chronic Kidney Disease Epidemiology Collaboration); FS, fractional shortening; IVS, interventricular septum; LAD, left atrium diameter; LVDd, left ventricular end-diastolic dimension; LVDs, left ventricular end-systolic dimension; LVEF, left ventricular ejection fraction; LVH, left ventricular hypertrophy; LVM, left ventricular mass (g) = 0.8 * 10.4 * ([left ventricular end-diastolic dimension + left ventricular posterior wall + interventricular septum]^3^- left ventricular end-diastolic dimension^3^) + 0.6; LVMI, left ventricular mass index (g/m^2^) = left ventricular mass/body surface area (= 0.0061 * height [cm] + 0.0128 * weight [kg] - 0.1529); Lp-PLA_2_, lipoprotein-associated phospholipase 2; LVPW, left ventricular posterior wall; n, number; NA, not applicable; NT-proBNP, N-Terminal pro hormone B-type Natriuretic Peptid; RAD, right atrial diameter; RVDd, right ventricular end-diastolic diameter; RWT, relative wall thickness =2 * left ventricular posterior wall/left ventricular end-diastolic dimension. Cardiovascular disease was confirmed if there was a definite manifestation of coronary heart disease, stroke (including transient ischemic attack) or congestive heart failure. Chronic kidney disease was defined as estimated glomerular filtration rate < 60 mL/min/1.73m^2^. Left ventricular hypertrophy was defined as left ventricular mass index ≥115 g/m^2^ for men and ≥95 g/m^2^ for women.

**Figure 4:**
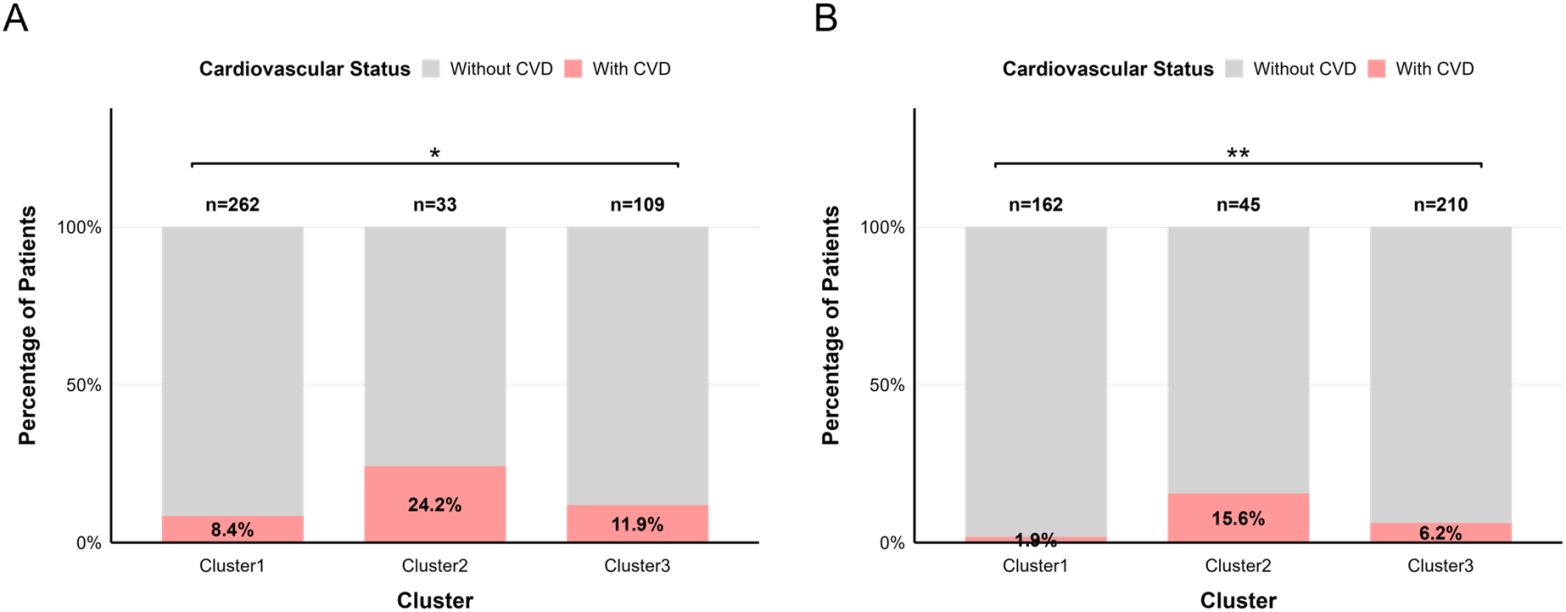
Cardiovascular disease incidence across clusters in RIA individuals. **(A)** Discovery cohort. **(B)** Validation cohort. CVD, cardiovascular disease. ^*^P < 0.05; ^**^P < 0.01.

**Figure 5:**
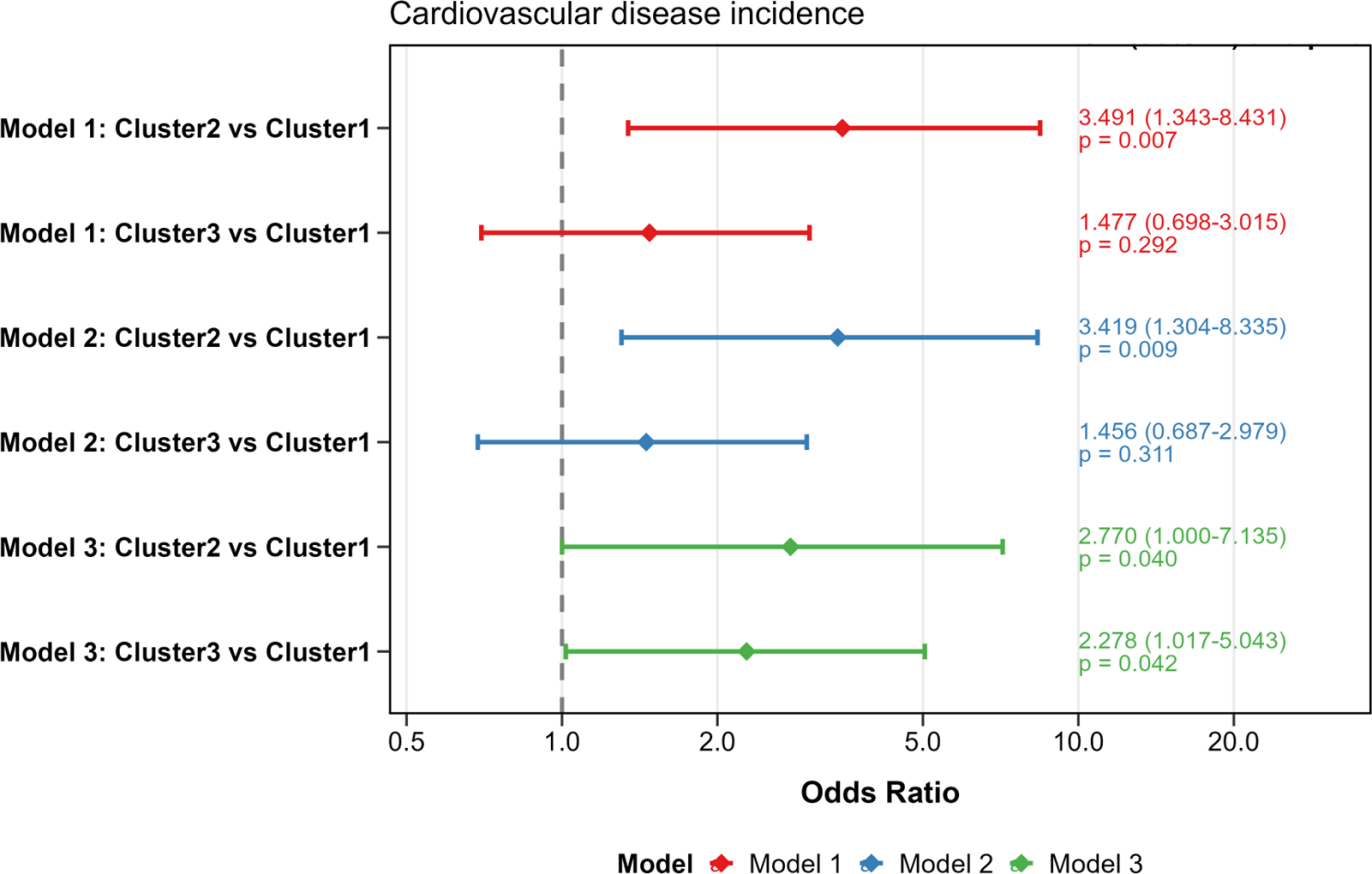
The associations of different clusters and cardiovascular disease incidence in the discovery cohort. Model 1 served as the baseline with no covariate adjustments. Model 2 included adjustments from Model 1, plus factors such as smoking status and alcohol use. Model 3 built upon Model 2 by additionally adjusting for statin use and diabetes treatment. In all models, cluster 1 was used as the reference group.

As shown in **Figure 4**, cardiovascular disease incidence was highest in cluster 2 (n=8, 24.2%), followed by cluster 3 (n=13, 11.9%), and lowest in cluster 1 (n=22, 8.4%; *P* = 0.025). We used multinomial logistic regression to compare the odds of cardiovascular disease incidence across the three clusters. Model 1 was sequentially adjusted in two stages by including different sets of covariates. Results from both stages of adjustment showed that the odds of cardiovascular disease incidence were significantly higher in cluster 2 (odds ratio = 2.770, 95% CI: 1.000-7.135; *P* = 0.002) and cluster 3 (odds ratio = 2.278, 95% CI: 1.017-5.043; *P* = 0.042) compared to cluster 1 (reference group) (**Figure 5**, **Supplementary Table S4**).

### External validation of clinical characteristics and cardiac outcomes of distinct clusters

Data from validation cohort identified 3 clusters with clinical characteristics similar to those in discovery cohort: cluster 1 (n=162, 38.8%), cluster 2 (n=45, 10.8%) and cluster 3 (n=210, 50.4%). The t-SNE analysis exhibited and verified the polarized distributions of patient characteristics across the clusters (**Supplementary Figure S4A**). The distribution of aberrant clustering features across different clusters is shown in **Supplementary Figures S4C, S4D**. Patients in cluster 1 were the youngest (*P*<0.001), had the highest proportion of female (*P*<0.001), the lowest systolic (*P*<0.001) and diastolic blood pressure (*P*<0.001), and exhibited the least severe metabolic dysfunction relative to the other 2 clusters. Patients in cluster 2 showed the most pronounced metabolic abnormalities, with the highest levels of BMI (*P*<0.001), total cholesterol (*P*<0.001), triglycerides (*P*<0.001), and fasting blood glucose (*P*<0.001), as well as the highest prevalence of obesity (*P*<0.001), dyslipidemia (*P*<0.001), diabetes (*P*<0.001) and the greatest use of antidiabetic therapy (*P*<0.001). Patients in cluster 3 exhibited the highest plasma aldosterone concentrations (*P* = 0.020) and the greatest use of statins (*P*<0.001) (**Supplementary Figure S**, **Supplementary Table S3**).

Cluster 2 exhibited the highest cardiovascular disease incidence (n=7, 15.6%) in **Figure 4**, followed by cluster 3 (n=13, 6.2%), with cluster 1 showing the lowest rate (n=3, 1.9%; *P*<0.01). Next, we analyzed baseline cardiovascular parameters across the defined RIA clusters in the validation cohort. These findings were consistent with those from the discovery cohort. Cluster 1 exhibited the least severe cardiac structural abnormalities at baseline, assessed by echocardiographic measures, compared with clusters 2 and 3. Patients in cluster 2 demonstrated the most pronounced left ventricular cardiac remodeling (**Table 2**).

**Table 2:** Echocardiographic parameters in different clusters of patients with RIA in the validation cohort.

Variables	Total (n=403)	Cluster 1 (n=160)	Cluster 2 (n=39)	Cluster 3 (n=204)	Overall *P* value	Pairwise Comparison (*P* value)		
						Cluster 1 vs Cluster 2	Cluster 1 vs Cluster 3	Cluster 2 vs Cluster 3
LAD, mm	27.68 ± 4.59	25.52 ± 3.65	31.64 ± 5.32	28.49 ± 4.26	<0.001	<0.001	<0.001	<0.001
IVSd, mm	9.00 (8.30-10.00)	8.35 (7.70-9.00)	10.30 (9.40-11.05)	9.40 (8.60-10.20)	<0.001	<0.001	<0.001	<0.001
IVSs, mm	13.10 (12.10-14.40)	12.40 (11.50-13.38)	14.35 (13.23-15.85)	13.50 (12.35-14.90)	<0.001	<0.001	<0.001	0.005
LVPWd, mm	9.10 (8.20-9.70)	8.35 (7.70-8.90)	10.25 (9.28-10.70)	9.30 (8.55-9.90)	<0.001	<0.001	<0.001	<0.001
LVPWs, mm	13.84 ± 1.89	13.02 ± 1.65	15.03 ± 1.69	14.23 ± 1.86	<0.001	<0.001	<0.001	0.023
LVDd, mm	49.62 ± 4.06	48.40 ± 3.73	51.40 ± 3.69	50.24 ± 4.12	<0.001	<0.001	<0.001	0.282
LVDs, mm	31.94 ± 3.31	31.11 ± 3.13	33.54 ± 2.89	32.28 ± 3.37	<0.001	<0.001	0.002	0.075
LVEDV, mL	117.29 ± 22.16	110.54 ± 19.86	126.95 ± 20.70	120.73 ± 22.75	<0.001	<0.001	<0.001	0.294
LVESV, mL	41.48 ± 10.32	38.88 ± 9.58	46.41 ± 9.38	42.56 ± 10.55	<0.001	<0.001	0.002	0.082
LVSV, mL	75.82 ± 13.99	71.66 ± 12.40	80.61 ± 13.72	78.17 ± 14.43	<0.001	<0.001	<0.001	0.917
LVEF, %	64.85 ± 4.31	65.03 ± 4.31	63.58 ± 4.07	64.96 ± 4.34	0.149	NA	NA	NA
LVM, g	162.59 ± 43.92	140.18 ± 34.30	202.88 ± 43.14	172.48 ± 41.76	<0.001	<0.001	<0.001	<0.001
LVMI, g/m^2^	82.84 (72.35-95.64)	77.22 (68.81-89.93)	86.15 (77.00-100.57)	85.54 (75.96-100.19)	<0.001	0.010	<0.001	1.000
LVH, %	39 (9.7%)	14 (8.8%)	4 (10.3%)	21 (10.3%)	0.852	NA	NA	NA

Quantitative normally distributed variables are expressed as means with standard deviations and non-normally distributed variables are shown as medians and interquartiles. Categorical variables are presented as absolute numbers and percentages. *P* values are calculated using Chi-square and Fisher’s exact tests or ANOVA followed by Bonferroni tests or Kruskal-Wallis tests followed by pairwise comparisons as appropriate. *P*<0.05 was considered significant. IVSd: interventricular septum diastolic thickness; IVSs: interventricular septum systolic thickness; LAD, left atrium diameter; LVDd, left ventricular end-diastolic dimension; LVDs, left ventricular end-systolic dimension; LVEDV: left ventricular end-diastolic volume; LVEF, left ventricular ejection fraction; LVESV: left ventricular end-systolic volume; LVH, left ventricular hypertrophy; LVM, left ventricular mass (LVM (g) = 0.8 * 10.4 * [(LVDd + LVPW + IVS)^3^- LVDd^3^] + 0.6); LVMI, left ventricular mass index (LVMI (g/m^2^) = LVM/body surface area (BSA = 0.0061 * height (cm) + 0.0128 * weight (kg) - 0.1529); LVPWd: left ventricular posterior wall diastolic thickness; LVPWs: left ventricular posterior wall systolic thickness; LVSV: left ventricular stroke volume; n, number; NA, not applicable. LVH was defined as LVMI≥115 g/m^2^ for men and ≥95 g/m^2^ for women.

**Figure 6:**
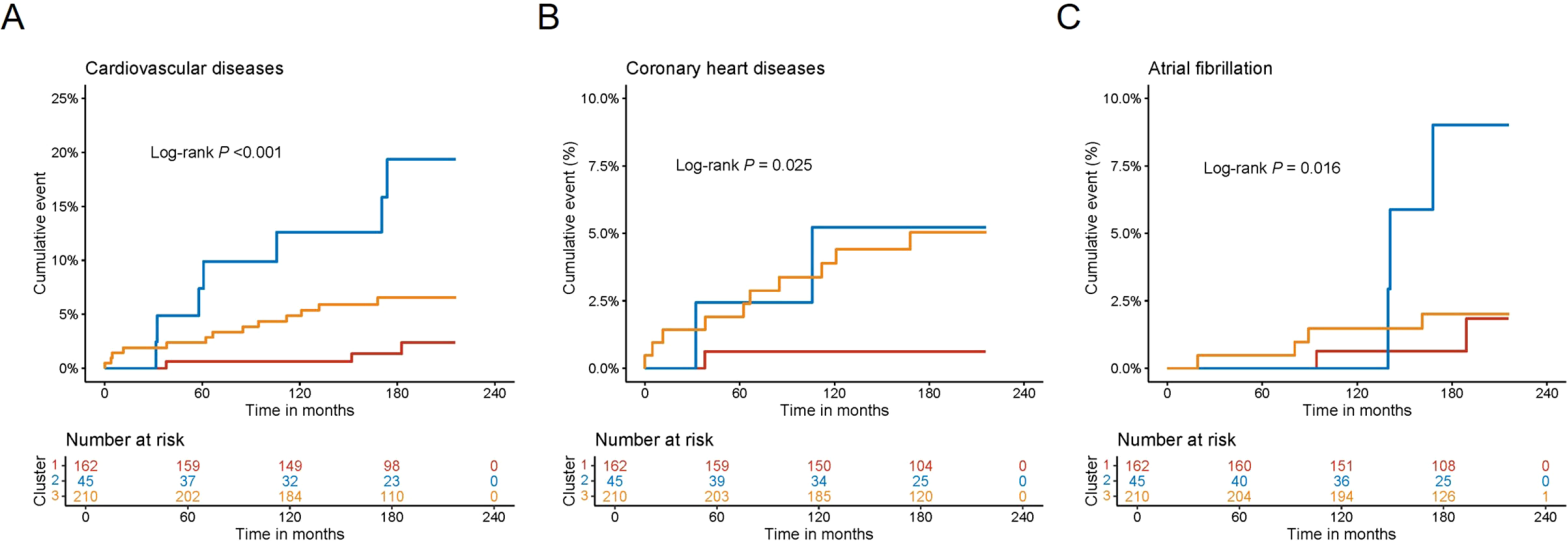
Cumulative incidences of cardiovascular events across 3 clusters in the validation cohort. Kaplan-Meier curves were plotted for the cumulative incidence of cardiovascular events among 3 clusters. The log-rank test was used to assess differences among clusters. **(A)** Cardiovascular disease. **(B)** Coronary heart diseases. **(C)** Atrial fibrillation.

During a median follow-up of 5543 [4899-6033] days, 23 cardiovascular disease events, 13 coronary heart disease events, and 10 atrial fibrillation events occurred in the validation cohort. The Kaplan-Meier curves revealed that the cumulative incidence of adverse cardiovascular events in cluster 2 were higher than those in cluster 1 and cluster 3 (cardiovascular disease, log rank: *P*<0.001; coronary heart disease, log rank: *P* = 0.025; atrial fibrillation, log rank: *P* = 0.016) (**Figure 6**). To further examine the relationship between cluster classification and cardiovascular outcomes, Cox regression models were employed using patients from cluster 1 as the reference. Multivariate Cox proportional hazards models adjusted for smoking status, alcohol use, statin use and diabetes treatment demonstrated that patients in clusters 2 had a significantly higher risk of developing cardiovascular disease (hazard ratio = 11.425, 95% CI: 2.218-58.844; *P* = 0.004), chronic heart failure (hazard ratio = 7.632, 95% CI: 1.563-42.673; *P* = 0.043) and atrial fibrillation (hazard ratio = 12.879, 95% CI: 1.325-125.158; *P* = 0.028) compared with the other 2 clusters (**Table 3**). The risk of cardiovascular disease (hazard ratio = 5.028, 95% CI: 1.124-22.494, *P* = 0.035) was also higher in cluster 3 than in cluster 1 (**Table 3**).

**Table 3:** Cox proportional hazard ratios for cardiovascular events according to clusters in the validation cohort.

Cluster	Model 1		Model 2		Model 3	
	HR (95% CI)	*P* value	HR (95% CI)	*P* value	HR (95% CI)	*P* value
Cardiovascular disease						
Cluster 1	1 (ref.)	–	1 (ref.)	–	1 (ref.)	–
Cluster 2	15.086 (3.133, 72.649)	<0.001	14.554 (3.018, 70.184)	<0.001	11.425 (2.218, 58.844)	0.004
Cluster 3	5.225 (1.179, 23.157)	0.029	4.955 (1.114, 22.047)	0.036	5.028 (1.124, 22.494)	0.035
Chronic heart failure						
Cluster 1	1 (ref.)	–	1 (ref.)	–	1 (ref.)	–
Cluster 2	8.236 (0.747, 90.852)	0.085	8.049 (0.728, 88.985)	0.089	7.632 (1.563, 42.673)	0.043
Cluster 3	7.962 (1.019, 62.202)	0.048	7.901 (1.005, 62.095)	0.049	4.236 (0.834, 37.548)	0.084
Atrial fibrillation						
Cluster 1	1 (ref.)	–	1 (ref.)	–	1 (ref.)	–
Cluster 2	12.838 (1.335, 123.445)	0.027	12.088 (1.255, 116.444)	0.031	12.879 (1.325, 125.158)	0.028
Cluster 3	3.173 (0.355, 28.397)	0.302	2.670 (0.297, 23.984)	0.381	1.675 (0.165, 17.014)	0.663

We used a Cox proportional hazards model to compare the risk of cardiovascular outcomes among the 3 clusters. A hazard ratio greater than 1 indicates a higher incidence of cardiovascular events in cluster 2 or 3 than in cluster 1, whereas a hazard ratio less than 1 indicates a lower incidence. Model 1 served as the baseline with no covariate adjustments. Model 2 included adjustments from Model 1, plus factors such as smoking status and alcohol use. Model 3 built upon Model 2 by additionally adjusting for statin use and diabetes treatment. In all models, cluster 1 was used as the reference group. CI, confidence interval; HR, hazard ratio; ref., reference.

## Discussion

Using cross-sectional data from our registry cohort and longitudinal data from Gen III cohort, we identified a new clustering of individuals with RIA, which is associated with varying degree of target organ damage and different risks of adverse cardiovascular events. This classification provides valuable insights for guiding therapeutic decisions. We incorporated variables reflective of key clinical features of RIA that are monitored in patients, ensuring that this clustering approach can be applied to clinical practices.

The consistency of clustering results between patients suspected of endocrine hypertension in our registry cohort and the general population enrolled in Gen III study suggests that these clusters are stable and potentially mechanistically distinct, rather than merely reflecting different stages of RIA. Physiologically, renin-independent aldosterone secretion is a response to extracellular hyperkalemia (9), whereas pathological renin-independent secretion is driven by autonomous aldosterone secretion (9) that is typically associated with adrenal abnormalities (50, 51) and aldosterone-driver mutations (52). The phenotypic profile of cluster 3, characterized by the highest aldosterone levels, is consistent with a pathophysiology that may involve adrenal abnormalities and aldosterone-driver mutations (52). We also identified a subgroup of patients with RIA (cluster 2) who exhibit most severe metabolic syndrome but milder aldosterone secretion compared with cluster 3. Although the mechanisms underlying this pattern remain uncertain, metabolic factors could play a role in modulating aldosterone secretion in these individuals. Previous studies have suggested that leptin may stimulate aldosterone production (53), and that lipid abundance can affect steroidogenesis in aldosterone-producing cells by contributing to phospholipid formation (54), altering oxidative stress (31, 55), and acting as lipid second messengers (56) that regulate steroidogenic enzyme activity and transcription. These observations raise the possibility that the dysregulated lipid metabolism observed in cluster 2 may contribute to renin-independent aldosterone secretion. This interpretation is compatible with previous studies showing a higher prevalence of obesity in patients with bilateral adrenal hyperplasia or nonclassical adrenal histopathology, which are subtypes associated with a milder phenotype of primary aldosteronism, compared with those with aldosterone-producing adenomas or classical histopathology in which aldosterone-driver mutations account for approximately 95% (31, 57, 58). The characteristics of cluster 1 shown in both cohorts is consistent with previous reports suggesting a possible association of elevated aldosterone-to-renin ratio with older age, female and chronic kidney disease (59, 60).

Previous population-based studies have reported an increased risk of cardiovascular disease (4, 12, 13), and a higher prevalence of chronic kidney disease (5) and metabolic dysfunction-associated steatotic liver disease (6) in patients with RIA compared with those with renin-dependent aldosteronism. The association between aldosterone and adverse cardiac remodeling or long-term cardiovascular risk has been demonstrated in both normotensive and hypertensive persons across different age groups (12, 13, 17, 61). In line with these findings, our study revealed cardiac structural alteration and an elevated cardiovascular risk in cluster 3 which exhibited higher aldosterone concentrations compared with cluster 1. It has been suggested that the heightened cardiovascular risk in RIA or with increased aldosterone secretion is likely due to enhanced MR activity, as supported by previous studies showing a positive correlation between aldosterone concentrations and MR activation (4, 11). These findings support the use of MR antagonists in patients with RIA to mitigate cardiovascular risk.

However, hypertension practice guidelines at present do not provide recommendations on the treatment of patients with RIA who do not meet the diagnostic criteria for primary aldosteronism (62). Our clustering results suggest that the subgroup of RIA characterized by a high aldosterone concentration (cluster 3) may benefit most from treatment with MR antagonists and potentially aldosterone synthase inhibitors which showed to lower blood pressure by reducing aldosterone production in clinical trials (63). For the subgroup of RIA who exhibit severe metabolic abnormalities and the highest cardiovascular risk (cluster 2), treatment should prioritize both metabolic and cardiovascular protection. While angiotensin-converting enzyme inhibitors and angiotensin receptor blockers have demonstrated benefits in improving insulin sensitivity (26), preserve pancreatic beta-cell function (22, 23) and mitigate metabolic complications (24, 25), their antihypertensive effects might be limited in RIA (18). Among MR antagonists, the steroidal agents spironolactone and eplerenone have metabolic limitations. Spironolactone has been associated with an increase in Hemoglobin A1c, possibly due to its non-selective action which raises cortisol level and subsequently activates glucocorticoid receptors (27). Eplerenone has not demonstrated significant benefits for glucose and lipid metabolism (28, 29). In contrast, finerenone, a non-steroidal MR antagonist with broader distribution in extra-renal tissues (64), shows more potent anti-inflammatory and antifibrotic effects than steroidal MR antagonists (65, 66). Notably, the cardiovascular benefits of finerenone are more pronounced in patients with a higher BMI (67), and it has also been shown to reduce diabetes incidence in patients with heart failure (30). Investigations should be warranted to explore if finerenone, which is currently used in patients with chronic kidney disease and type 2 diabetes (68), can improve blood pressure (69) and cardiovascular health (70) in RIA, especially in cluster 2, where metabolic dysfunction is most severe.

## Strength and limitations

The key strengths of the current study included the relatively large sample size of patients with RIA, which is comparable to that reported in previous large-scale population studies, the detailed evaluation of target organ damage in our registry cohort and the confirmation of the clustering results in 2 independent populations. Although patients in the discovery and validation cohorts exhibited significantly different clinical characteristics, the overall consistency of the clustering results indicates the reliability of our findings and supports the concept that RIA may comprise mechanistically distinct subgroups warranting differentiated therapeutic strategies. Nonetheless, several limitations should be acknowledged. First, the presence of confounders such as interfering medications, patient’s posture at blood sampling and electrolyte imbalance in the Gen III study, combined with the known intraindividual variability of aldosterone and renin levels (71), may have introduced misclassification bias due to the reliance on a single measurement. Second, our clustering results cannot be confidently interpreted as representing different etiologies of RIA, as we did not collect data on the cortisol levels after 1 mg dexamethasone test, which is the more accurate indicator of autonomous cortisol production than baseline cortisol levels and may have influenced the clustering results. Third, the blood pressure measurements used in both cohorts were obtained from clinic visits, which may not provide the same level of precision as ambulatory blood pressure monitoring. Fourth, the limited number of outcome events in the Gen III cohort, together with the smaller sample size of the high-metabolic-risk cluster (cluster 2; n=45), contributed to wide confidence intervals in the Cox regression analysis and raised concerns about potential overfitting. Although our power calculation indicated sufficient power (e.g., 80% power to detect a hazard ratio ≥3.5 for cluster 2 versus cluster 1 in the Cox regression analysis), the precision of the risk estimates for this specific subgroup remains limited. Nevertheless, the reproducible identification of cluster 2 in both cohorts, its consistently severe metabolic phenotype, and its numerically higher cardiovascular event rates support its potential clinical relevance, warranting further validation in larger prospective studies. Fifth, the exclusion of overt primary aldosteronism diagnosis, unilateral secretion status, and hypertension duration as clinical variables for stratifying target organ damage may limit precision. These factors were omitted to ensure replication, as they were unavailable in the validation cohort. Nevertheless, the distribution of primary aldosteronism appeared consistent with the clinical profiles of each cluster, and future studies are warranted to determine whether incorporating these variables could further enhance stratification accuracy. Finally, it is important to acknowledge that although cardiovascular outcomes were assessed longitudinally in the validation cohort, the clustering analysis itself was derived exclusively from cross-sectional baseline data. RIA is a dynamic condition, and patient phenotypes may evolve over time in response to disease progression, therapeutic interventions, or lifestyle modifications. Accordingly, we cannot exclude the possibility that the identified clusters represent either stable pathophysiological traits or transient clinical states. Longitudinal studies with repeated measurements of the clustering variables will be essential to determine the temporal stability of these clusters and to elucidate how potential cluster transitions relate to changes in cardiovascular risk and treatment response.

## Conclusions and perspectives

In conclusion, our data suggested that integrating clinical variables from patients of RIA can effectively stratify subgroups with varying degree of target organ damage and long-term cardiovascular risks. Our findings indicate that metabolic dysfunction, rather than aldosterone excess, could be an important contributor to cardiovascular risk in RIA patients, though causal inference awaits confirmation in future prospective research. Given the reported association of renin-independent aldosterone secretion and cardiovascular complications (12, 13, 17, 61), clinical trials are warranted to evaluate the impact of early intervention with MR antagonists on long-term cardiovascular health outcome in patients with RIA. Our clustering highlights the need for additional focus in these clinical trials on patients with RIA with severe metabolic dysfunction to guide the optimal medication.

## Data Availability

The original contributions presented in the study are included in the article/**Supplementary Material**, further inquiries can be directed to the corresponding author/s.
