# Supplementary material for: Data-driven cluster analysis and external validation identify phenotypic subgroups in renin-independent aldosteronism with differential cardiovascular risk and therapeutic implications
Source: Front Endocrinol (Lausanne). 2025 Dec 15;16:1686480. doi: 10.3389/fendo.2025.1686480 (PMC12745265; doi:10.3389/fendo.2025.1686480)
Supplement: Supplementary file 1 [file DataSheet1.pdf]

Data-Driven Cluster Analysis and External Validation Identify Phenotypic Subgroups in Renin-Independent Aldosteronism with Differential Cardiovascular Risk and Therapeutic Implications

Yuqing Liu[1]†, Zhiheng Zhang[2]†, Haifeng Zhou[3], Yutong Yan[1], Mei Zhou[1], Cong Wang[1], Maoting Gao[1], Jun Tao[4], Meiling Bao[5], Tao Yang[1]*‡, Min Sun[1]*‡, Yuhong Yang[1]*‡

[1]Department of Endocrinology, the First Affiliated Hospital With Nanjing Medical University, Nanjing, China
[2]Division of Hepatobiliary and Transplantation Surgery, Department of General Surgery, Nanjing Drum Tower Hospital, Affiliated Hospital of Medical School, Nanjing University, Nanjing, China
[3]Department of Interventional Radiology, the First Affiliated Hospital With Nanjing Medical University, Nanjing, China
[4]Department of Urology, the First Affiliated Hospital With Nanjing Medical University, Nanjing, China
[5]Department of Pathology, the First Affiliated Hospital With Nanjing Medical University, Nanjing, China

†These authors have contributed equally to this work and shared first authorship.
‡These authors have contributed equally to this work and shared last authorship.

***Corresponding author:**
Dr. Yuhong Yang, Email: yuhong.yang@njmu.edu.cn; Department of Endocrinology, the First Affiliated Hospital With Nanjing Medical University, Guangzhou Road 300, 210029 Nanjing, China
Prof. Min Sun, Email: drsunm@njmu.edu.cn; Department of Endocrinology, the First Affiliated Hospital With Nanjing Medical University, Guangzhou Road 300, 210029 Nanjing, China
Prof. Tao Yang, Email: yangt@njmu.edu.cn; Department of Endocrinology, the First Affiliated Hospital With Nanjing Medical University, Guangzhou Road 300, 210029 Nanjing, China

**Table S1**	Clinical characteristics of patients with RIA in the discovery and validation cohorts	Page 3-4
**Table S2**	Baseline characteristics of 3 clusters derived from hierarchical cluster analysis in patients with RIA in the discovery cohort	Page 5-6
**Table S3**	Baseline characteristics of 3 clusters derived from hierarchical cluster analysis in patients with RIA in the validation cohort	Page 7-8
**Table S4**	The associations of different clusters and cardiovascular disease incidence in the discovery cohort	Page 9
**Figure S1**	Missing data pattern in the discovery cohort	Page 10
**Figure S2**	Correlation matrix of selected parameters	Page 11
**Figure S3**	Dendrogram showing hierarchical relationship among 3 clusters identified in the discovery cohort	Page 12
**Figure S4**	Visualization of clustering results and patient distribution in the validation cohort	Page 13
**Figure S5**	Prevalence of primary aldosteronism in different clusters in the discovery cohort	Page 14
**Figure S6**	Prevalence of unilateral primary aldosteronism in different clusters in the discovery cohort	Page 15
**Figure S7**	Clinical characteristics of patients with RIA stratified by different clusters in the validation cohort	Page 16

Table S1. Clinical characteristics of patients with RIA in the discovery and validation cohorts.

Variables	Total (n=821)	Discovery (n=404)	Validation (n=417)	*P* value
**Clustering variables**				
Age, year	46.66±12.70	52.92±12.44	40.59±9.65	<0.001
Sex, ref. female	395 (48.1%)	184 (45.5%)	211 (50.6%)	0.147
BMI, kg/m^2	26.06±4.60	25.42±3.63	26.69±5.31	<0.001
Systolic BP, mmHg	130.60±21.14	143.00±18.67	118.58±15.78	<0.001
Diastolic BP, mmHg	81.15±12.97	86.35±13.60	76.11±10.04	<0.001
PAC, pg/mL	151.00 [122.00-218.35]	190.42 [139.13-288.41]	133.00 [115.00-167.50]	<0.001
PRA, μg/L/h	0.50 [0.23-0.76]	0.28 [0.13-0.51]	0.69 [0.49-0.84]	<0.001
Cortisol, nmol/L	326.11 [240.10-435.32]	276.35 [214.80-355.30]	384.60 [287.21-493.31]	<0.001
TC, mmol/L	4.66±0.95	4.59±1.05	4.74±0.83	0.032
TG, mmol/L	1.12 [0.79-1.66]	1.31 [0.93-1.82]	0.96 [0.71-1.37]	<0.001
FBG, mmol/L	5.06 [4.68-5.44]	4.87 [4.41-5.35]	5.17[4.94-5.50]	<0.001
FINS, pmol/L	37.08 [21.60-67.59]	63.80 [39.03-90.88]	22.92 [17.25-34.50]	<0.001
**Other variables**				
Current smoker, %	131 (16.0%)	73 (18.1%)	58 (13.9%)	0.104
Alcohol user, %	295 (35.9%)	70 (17.3%)	225 (54.0%)	<0.001
Obesity, %	175 (21.3%)	87 (21.5%)	88 (21.1%)	0.880
Dyslipidemia, %	358 (43.6%)	181 (44.8%)	177 (42.4%)	0.496
Use of statin, %	109 (13.3%)	92 (22.8%)	17 (4.1%)	<0.001
DM, %	100 (12.2%)	94 (23.3%)	6 (1.4%)	<0.001
Treated for DM, %	78 (9.5%)	73 (18.1%)	5 (1.2%)	<0.001
ARR	34.05 [19.66-87.52]	81.83 [36.04-169.83]	21.44 [15.85-31.78]	<0.001

Quantitative normally distributed variables are expressed as means with standard deviations and non-normally distributed variables are shown as medians (interquartile ranges). Categorical variables are presented as absolute numbers (%). *P* values are calculated using Chi-square and Fisher's exact tests or Student's t-test or Mann-Whitney U test as appropriate. *P*<0.05 is considered significant. ARR, aldosterone-to-renin ratio; BMI, body mass index; BP, blood pressure; DM, diabetes mellitus; FBG, fasting blood glucose; FINS, fasting insulin; h, hour; n, number; NA, not applicable; PAC,

plasma aldosterone concentration; PRA, plasma renin activity; ref., reference; TC, total cholesterol; TG, triglycerides.

Table S2. Baseline characteristics of 3 clusters derived from hierarchical cluster analysis in patients with RIA in the discovery cohort.

Variables	Total (n=404)	Cluster 1 (n=262)	Cluster 2 (n=33)	Cluster 3 (n=109)	Overall *P* value	Pairwise Comparison (*P* value)		
						Cluster 1 vs Cluster 2	Cluster 1 vs Cluster 3	Cluster 2 vs Cluster 3
**Clustering variables**								
Age, year	52.92±12.44	57.32±10.26	54.09±13.39	42.00±10.10	<0.001	0.290	<0.001	<0.001
Sex, ref. female	184 (45.5%)	122 (46.6%)	18 (54.5%)	44 (40.4%)	0.306	NA	NA	NA
BMI, kg/m^2	25.42±3.63	24.61±3.07	29.94±4.02	25.98±3.67	<0.001	<0.001	0.001	<0.001
Systolic BP, mmHg	143.00±18.67	137.20±16.99	149.00±17.11	155.13±16.56	<0.001	0.001	<0.001	0.206
Diastolic BP, mmHg	86.35±13.60	81.36±10.96	85.67±13.21	98.55±11.74	<0.001	0.123	<0.001	<0.001
PAC, pg/mL	190.42 [139.13-288.41]	154.90 [123.17-280.00]	153.05 [109.51-215.00]	192.58 [135.00-281.70]	0.009	0.399	0.057	0.018
PRA, µg/L/h	0.28 [0.13-0.51]	0.26 [0.15-0.61]	0.27 [0.11-0.49]	0.28 [0.10-0.35]	0.353	NA	NA	NA
Cortisol, nmol/L	276.35 [214.80-355.30]	283.60 [224.30-357.10]	294.45 [213.13-336.18]	218.00 [196.00-257.50]	0.283	NA	NA	NA
TC, mmol/L	4.59±1.05	4.63±1.16	4.54±0.67	4.53±0.87	0.664	NA	NA	NA
TG, mmol/L	1.31 [0.93-1.82]	1.20 [0.80-1.88]	1.96 [1.36-2.27]	1.36 [1.06-1.76]	0.001	0.001	0.566	0.022
FBG, mmol/L	4.87 [4.41-5.35]	4.86 [4.54-5.28]	6.50 [4.68-9.61]	4.60 [4.36-5.30]	<0.001	<0.001	1.000	<0.001
FINS, pmol/L	63.80 [39.03-90.88]	63.60 [39.85-75.10]	113.95 [60.58-183.93]	60.20 [31.00-73.20]	<0.001	<0.001	0.135	<0.001
**Other variables**								
Current smoker, %	73 (18.1%)	43 (16.4%)	9 (27.3%)	21 (19.3%)	0.276	NA	NA	NA
Alcohol user, %	70 (17.3%)	45 (17.2%)	5 (15.2%)	20 (18.3%)	0.914	NA	NA	NA
Obesity, %	87 (21.5%)	35 (13.4%)	21 (63.6%)	31 (28.4%)	<0.001	<0.001	0.001	<0.001
Dyslipidemia, %	181 (44.8%)	130 (49.6%)	18 (54.5%)	33 (30.3%)	0.001	0.594	<0.001	0.011
Use of statin, %	92 (22.8%)	68 (26.0%)	12 (36.4%)	12 (11.0%)	0.001	0.205	0.001	<0.001
DM, %	94 (23.3%)	65 (24.8%)	14 (42.4%)	15 (13.8%)	0.002	0.031	0.018	<0.001

Variables	Total (n=404)	Cluster 1 (n=262)	Cluster 2 (n=33)	Cluster 3 (n=109)	Overall *P* value	Pairwise Comparison (*P* value)		
						Cluster 1 vs Cluster 2	Cluster 1 vs Cluster 3	Cluster 2 vs Cluster 3
Treated for DM, %	73 (18.1%)	50 (19.1%)	12 (36.4%)	11 (10.1%)	0.002	0.022	0.033	<0.001
ARR	81.83 [36.04-169.83]	74.17 [27.09-138.39]	60.02 [32.02-141.44]	94.75 [45.58-167.76]	0.042	1.000	0.036	0.743
Duration HTN, years	7.00 [3.00-11.00]	8.00 [3.00-15.00]	8.00 [2.00-20.00]	4.00 [1.63-10.00]	<0.001	1.000	<0.001	0.091

Quantitative normally distributed variables are expressed as means with standard deviations and non-normally distributed variables are shown as medians and interquartiles. Categorical variables are presented as absolute numbers and percentages. *P* values are calculated using Chi-square and Fisher's exact tests or ANOVA followed by Bonferroni tests or Kruskal-Wallis tests followed by pairwise comparisons as appropriate. $P<0.05$ was considered significant. ARR, aldosterone-to-renin ratio; BMI, body mass index; BP, blood pressure; DM, diabetes mellitus; FBG, fasting blood glucose; FINS, fasting insulin; h, hour; HTN, hypertension; n, number; NA, not applicable; PAC, plasma aldosterone concentration; PRA, plasma renin activity; ref., reference; RIA, renin-independent aldosteronism; TC, total cholesterol; TG, triglycerides.

Table S3. Baseline characteristics of 3 clusters derived from hierarchical cluster analysis in patients with RIA in the validation cohort.

Variables	Total (n=417)	Cluster 1 (n=162)	Cluster 2 (n=45)	Cluster 3 (n=210)	Overall *P* value	Pairwise Comparison (*P* value)		
						Cluster 1 vs Cluster 2	Cluster 1 vs Cluster 3	Cluster 2 vs Cluster 3
**Clustering variables**								
Age, year	40.59±9.65	35.11±8.16	45.56±8.93	43.74±8.87	<0.001	<0.001	<0.001	0.602
Sex, ref. female	211 (50.6%)	122 (75.3%)	12 (5.7%)	77 (36.5%)	<0.001	<0.001	<0.001	0.202
BMI, kg/m^2	26.69±5.31	23.83±3.68	35.07±6.65	27.10±3.84	<0.001	<0.001	<0.001	<0.001
Systolic BP, mmHg	118.58±15.78	106.86±8.34	139.31±20.03	123.18±11.71	<0.001	<0.001	<0.001	<0.001
Diastolic BP, mmHg	76.11±10.04	69.39±7.40	86.13±12.69	79.15±7.65	<0.001	<0.001	<0.001	<0.001
PAC, pg/mL	157.35±76.24	144.63±47.55	158.73±51.88	166.87±72.32	0.020	0.809	0.016	1.000
PRA, μg/L/h	0.69 [0.49-0.84]	0.68 [0.49-0.84]	0.70 [0.47-0.81]	0.70 [0.50-0.86]	0.848	NA	NA	NA
Cortisol, nmol/L	384.60 [287.21-493.31]	386.81 [281.62-497.17]	369.71 [284.04-452.75]	390.26 [291.49-507.45]	0.648	NA	NA	NA
TC, mmol/L	4.74±0.83	4.21±0.65	5.11±0.77	5.06±0.76	<0.001	<0.001	<0.001	1.000
TG, mmol/L	0.96 [0.71-1.37]	0.72 [0.56-0.94]	1.75 [1.42-2.70]	1.07 [0.83-1.43]	<0.001	<0.001	<0.001	<0.001
FBG, mmol/L	5.27±0.75	4.96±0.36	6.14±1.69	5.32±0.43	<0.001	<0.001	<0.001	<0.001
FINS, pmol/L	22.92 [17.25-34.50]	18.69 [15.17-24.17]	55.20 [44.49-80.34]	23.91 [19.25-34.04]	<0.001	<0.001	<0.001	<0.001
**Other variables**								
Current smoker, %	58 (13.9%)	27 (16.7%)	8 (17.8%)	23 (11.0%)	0.210	NA	NA	NA
Alcohol user, %	225 (54.0%)	97 (59.9%)	23 (51.1%)	105 (50.0%)	0.153	NA	NA	NA
Obesity, %	88 (21.1%)	10 (6.2%)	33 (73.3%)	45 (21.4%)	<0.001	<0.001	<0.001	<0.001
Dyslipidemia, %	177 (42.4%)	19 (11.7%)	31 (68.9%)	127 (60.5%)	<0.001	<0.001	<0.001	0.291
Use of statin, %	17 (4.1%)	0 (0%)	2 (4.4%)	15 (7.1%)	<0.001	0.046	<0.001	0.745

Variables	Total (n=417)	Cluster 1 (n=162)	Cluster 2 (n=45)	Cluster 3 (n=210)	Overall *P* value	Pairwise Comparison (*P* value)		
						Cluster 1 vs Cluster 2	Cluster 1 vs Cluster 3	Cluster 2 vs Cluster 3
DM, %	6 (1.4%)	0 (0%)	5 (11.1%)	1 (0.5%)	<0.001	<0.001	1.000	<0.001
Treated for DM, %	5 (1.2%)	0 (0%)	4 (8.9%)	1 (0.5%)	<0.001	0.002	1.000	0.004
ARR	21.44 [15.85-31.78]	19.96 [15.54-30.69]	22.42 [16.50-30.06]	22.45 [15.80-32.84]	0.552	NA	NA	NA

Quantitative normally distributed variables are expressed as means with standard deviations and non-normally distributed variables are shown as medians and interquartiles. Categorical variables are presented as absolute numbers and percentages. *P* values are calculated using Chi-square and Fisher's exact tests or ANOVA followed by Bonferroni tests or Kruskal-Wallis tests followed by pairwise comparisons as appropriate. *P*<0.05 was considered significant. ARR, aldosterone-to-renin ratio; BMI, body mass index; BP, blood pressure; DM, diabetes mellitus; FBG, fasting blood glucose; FINS, fasting insulin; h, hour; n, number; NA, not applicable; PAC, plasma aldosterone concentration; PRA, plasma renin activity; ref., reference; RIA, renin-independent aldosteronism; TC, total cholesterol; TG, triglycerides.

Table S4. The associations of different clusters and cardiovascular disease incidence in the discovery cohort.

Cardiovascular disease incidence			
	**OR**	**95%CI**	***P* value**
**Model 1**			
Cluster 1	1 (ref.)		
Cluster 2	3.491	1.343-8.431	0.007
Cluster 3	1.477	0.698-3.015	0.292
**Model 2**			
Cluster 1	1 (ref.)		
Cluster 2	3.419	1.304-8.335	0.009
Cluster 3	1.456	0.687-2.979	0.311
**Model 3**			
Cluster 1	1 (ref.)		
Cluster 2	2.770	1.000-7.135	0.040
Cluster 3	2.278	1.017-5.043	0.042

We used multinomial logistic regression model to compare the odds of cardiovascular disease incidences among the 3 clusters. A odds ratio greater than 1 indicates a higher incidence of cardiovascular diseases in cluster 2 or 3 than in cluster 1, whereas a odds ratio less than 1 indicates a lower incidence. Model 1 served as the baseline with no covariate adjustments. Model 2 included adjustments from Model 1, plus factors such as smoking status and alcohol use. Model 3 built upon Model 2 by additionally adjusting for statin use and diabetes treatment. In all models, cluster 1 was used as the reference group. CI, confidence interval; OR, odds ratio; ref., reference.

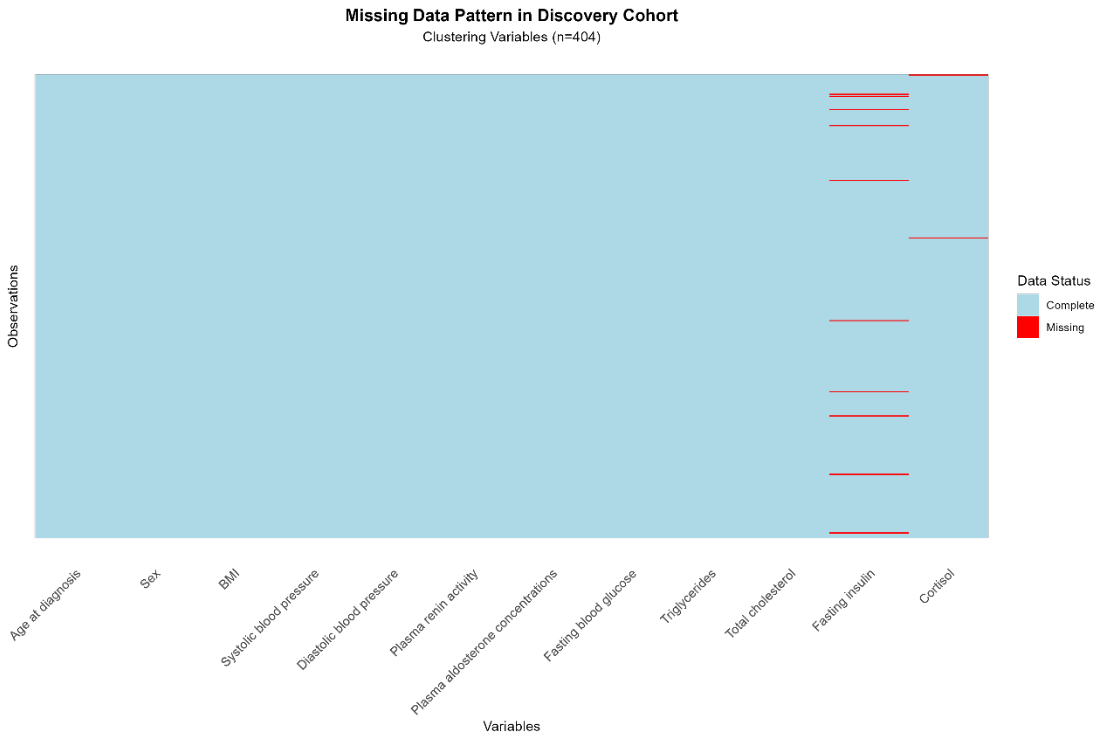

Figure S1. Missing data pattern in the discovery cohort.

The proportion of missing data for the 12 clustering variables was generally low, with fasting insulin having the highest proportion at 2.48% (10 missing values), followed by cortisol at 0.50% (2 missing values), while the remaining 10 variables had complete data (0% missing). BMI, body mass index.

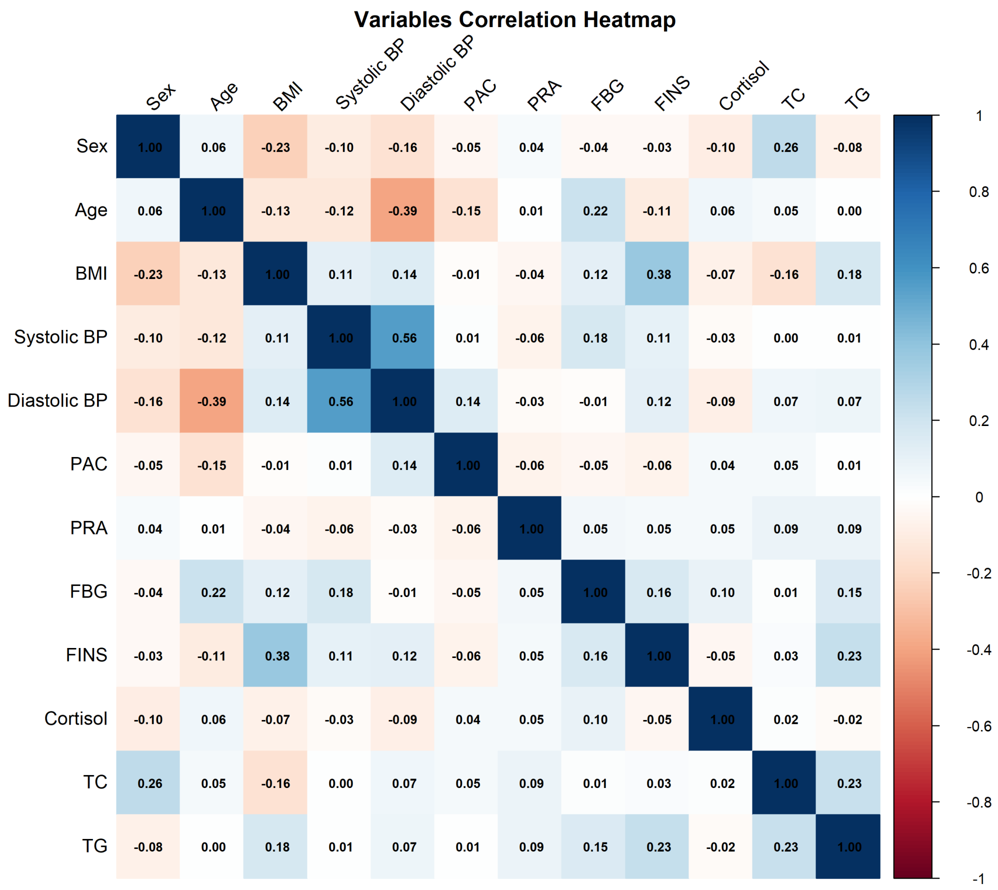

Figure S2. Correlation matrix of selected parameters.

Heatmap visualization depicting pairwise Pearson correlation coefficients (r) among the analyzed parameters. Overall correlations were weak, with the strongest positive correlation observed between systolic BP and diastolic BP (r = 0.56). Color intensity and direction (blue = positive, red = negative) correspond to the strength and sign of the correlation. BMI, body mass index; BP, blood pressure; FBG, fasting blood glucose; FINS, fasting insulin; PAC, plasma aldosterone concentration; PRA, plasma renin activity; TC, total cholesterol; TG, triglycerides.

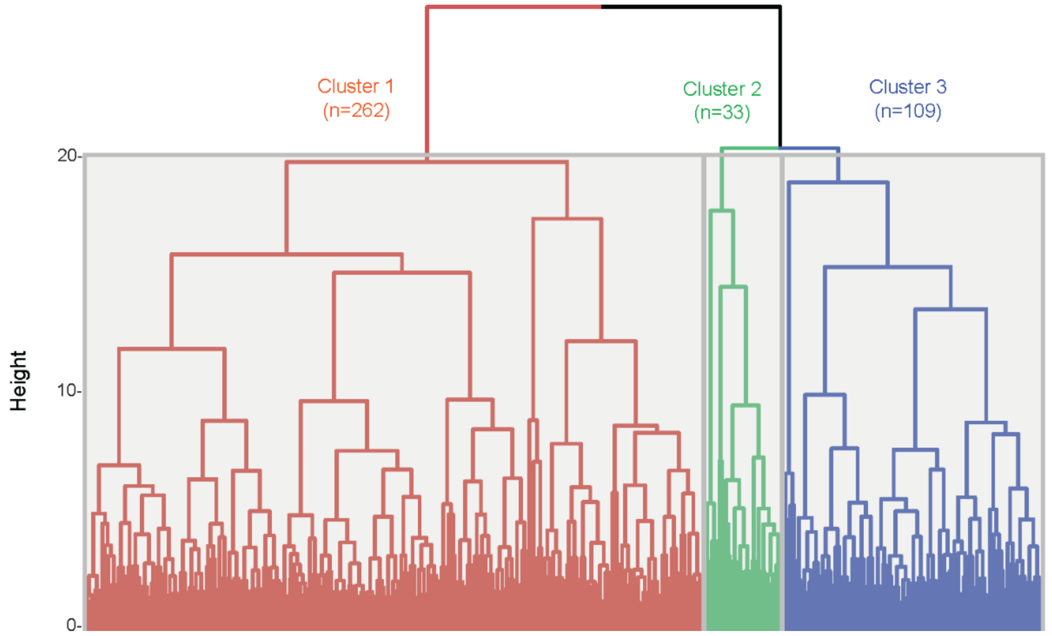

Figure S3. Dendrogram showing hierarchical relationship among 3 clusters identified in the discovery cohort.

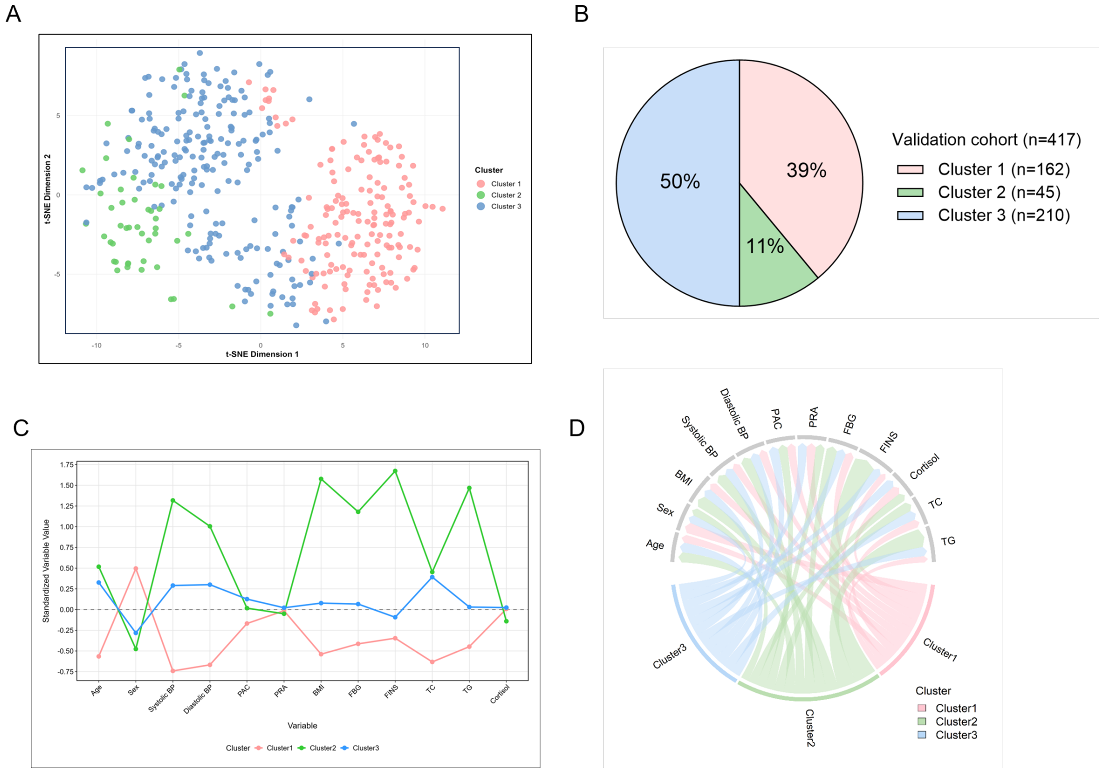

Figure S4. Visualization of clustering results and patient distribution in the validation cohort.

(A) t-Distributed Stochastic Neighbor Embedding (t-SNE) plot visualizing high-dimensional data. **(B)** Patient distribution across clusters. **(C)** Standardized values (z-scores) of selected variables by cluster. All continuous variables were scaled (mean = 0, SD = 1). **(D)** The ribbons connect an individual cluster to a variable if the group mean is greater or less than the overall mean for the entire cohort. BMI, body mass index; BP, blood pressure; FBG, fasting blood glucose; FINS, fasting insulin; PAC, plasma aldosterone concentration; PRA, plasma renin activity; RIA, renin-independent aldosteronism; TC, total cholesterol; TG, triglycerides.

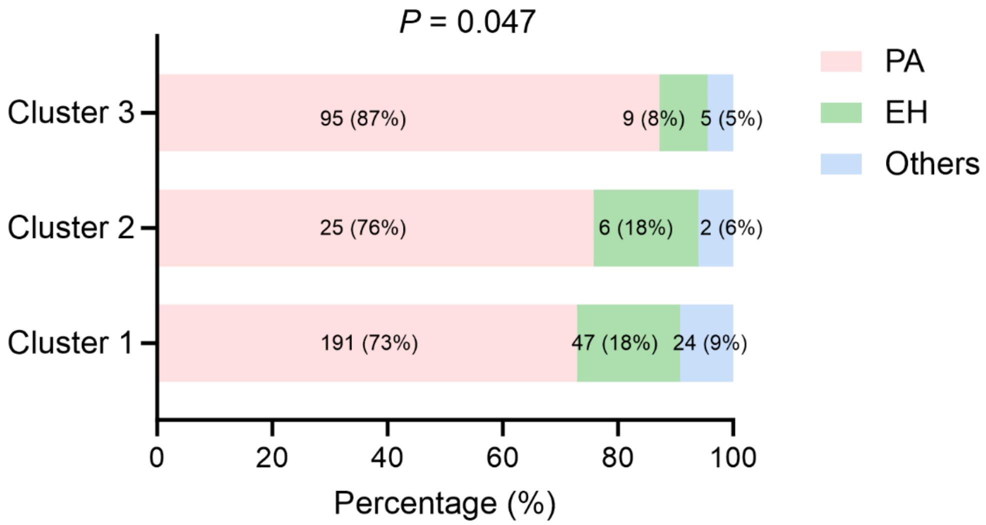

Figure S5. Prevalence of primary aldosteronism in different clusters in the discovery cohort.

EH, essential hypertension; PA, primary aldosteronism.

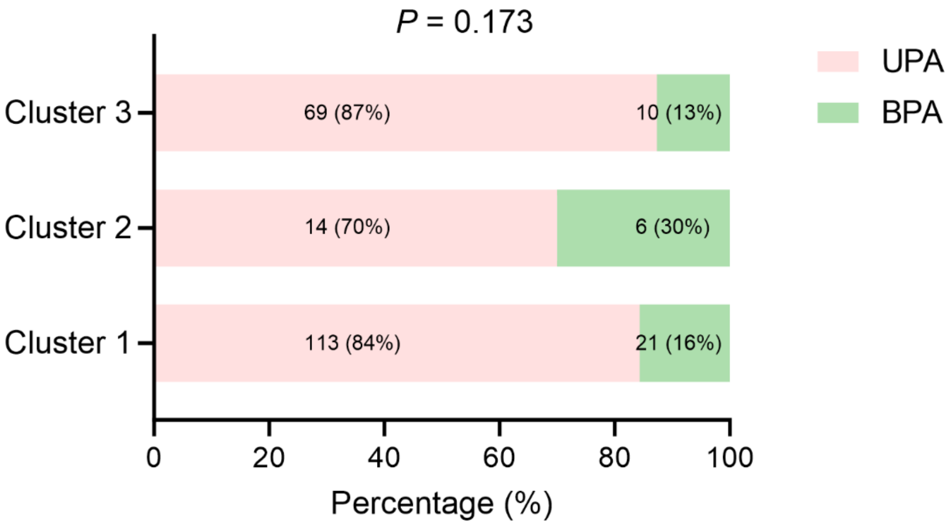

Figure S6. Prevalence of unilateral primary aldosteronism in different clusters in the discovery cohort.

BPA, bilateral primary aldosteronism; UPA, unilateral primary aldosteronism.

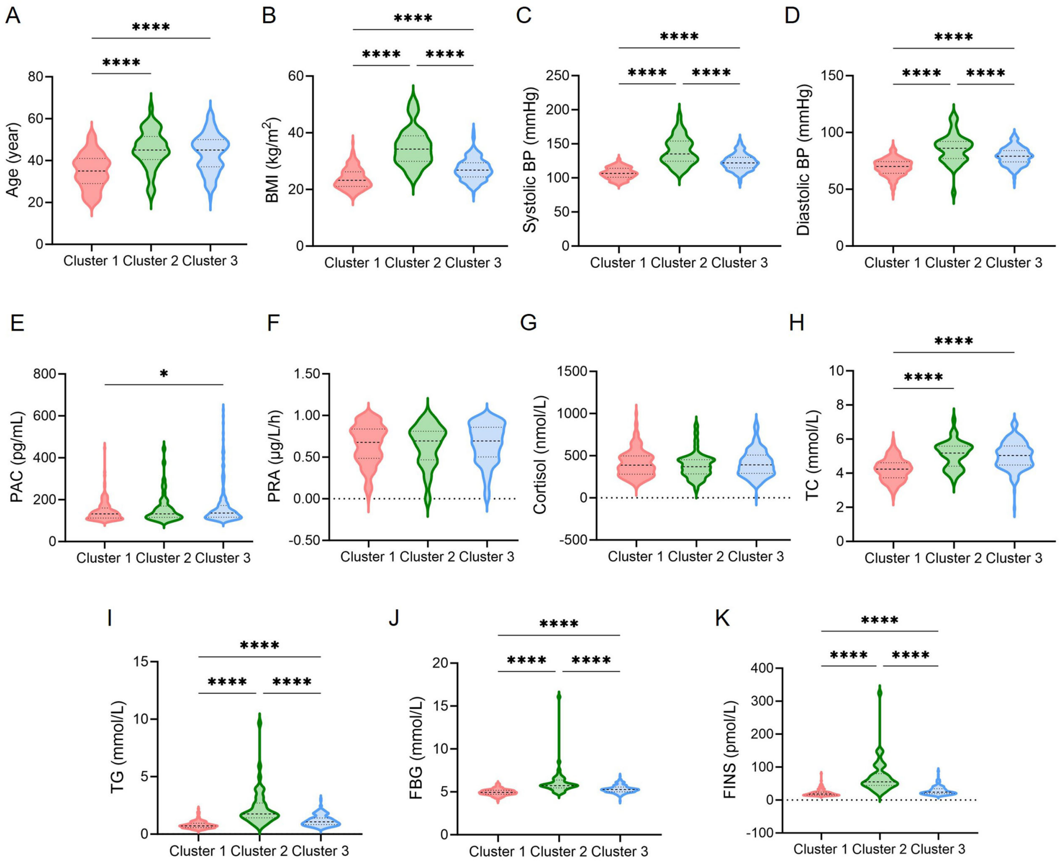

Figure S7. Clinical characteristics of patients with RIA stratified by different clusters in the validation cohort.

The distribution of **(A)** age, **(B)** BMI, **(C)** systolic blood pressure, **(D)** diastolic blood pressure, **(E)** plasma aldosterone concentration, **(F)** plasma renin activity, **(G)** cortisol, **(H)** total cholesterol, **(I)** triglycerides, **(J)** fasting blood glucose, and **(K)** fasting insulin in different clusters in the discovery cohort. BMI, body mass index; BP, blood pressure; FBG, fasting blood glucose; FINS, fasting insulin; h, hour; PAC, plasma aldosterone concentration; PRA, plasma renin activity; RIA, renin-independent aldosteronism; TC, total cholesterol; TG, triglycerides. $^{*}P < 0.05$; $^{****}P < 0.0001$.
